# Platform-specific digital media use and psychological distress among Turkish adults: evidence from a nationally representative, cross-sectional survey

**DOI:** 10.3389/fpubh.2026.1941229

**Published:** 2026-09-21

**Authors:** Aydın Aktay, Muhammed Veysel Bilici, Mehmet Veysel Karataş, Abdullah Yıldırmaz

**Affiliations:** 1 Department of Sociology, Faculty of Humanities and Social Sciences, Sakarya University, Sakarya, Türkiye; 2 Department of Sociology, Faculty of Arts and Sciences, Kırklareli University, Kırklareli, Türkiye; 3 Department of Philosophy and Religious Studies, Faculty of Theology, Siirt University, Siirt, Türkiye; 4 Public Relations and Publicity Program, Vocational School of Social Sciences, Batman University, Batman, Türkiye

**Keywords:** age, digital media use, loneliness, nationally representative survey, platform heterogeneity, psychological distress, social media, Türkiye

## Abstract

Digital media’s mental-health signature may depend on which platform a population uses, yet nationally representative evidence that distinguishes platforms is scarce, especially for upper-middle-income, Muslim-majority, non-Arab settings. We examined platform-specific associations between digital media use and psychological distress in the Turkish adult population, and whether age moderates them, using the 2024 Turkish General Social Survey, a probability-based, nationally representative survey of adults aged 18 and over (*N* = 2,615; analytical *N* = 1,478 after listwise deletion). Psychological distress was measured with a nine-item past-week negative-affect index informed by the Center for Epidemiologic Studies Depression Scale (CES-D), and unweighted hierarchical ordinary least squares regression with heteroskedasticity-consistent (HC3) robust standard errors modeled demographic, aggregate-exposure, platform-specific, age-interaction, and loneliness blocks, with a Johnson–Neyman procedure characterizing age moderation. Instagram use (*β* = +.066, *p* = .011) was associated with higher psychological distress and WhatsApp use (*β* = -.073, *p* = .007) with lower distress, whereas total internet time, TikTok, and YouTube showed no detectable association. Overall social-media time was likewise positively associated with distress (*β* = +.088, *p* = .016), but the platform contrasts, not aggregate exposure, survived the most demanding clustering and exposure-scale checks. The positive Instagram association declined with age and was statistically indistinguishable from zero by the early forties, a secondary finding that turned on whether loneliness entered the model (age-by-Instagram *β* = -.069, *p* = .014 with loneliness, *β* = -.060, *p* = .059 without). Adding loneliness — an affective covariate overlapping the outcome battery — raised explained variance to *R*^2^ = .359 (largely shared variance) while the platform and aggregate coefficients remained significant. In this national sample the platform types diverged: algorithm-driven image-platform use (Instagram) showed a small positive association with distress, interpersonal-messaging use (WhatsApp) a small inverse (protective-direction) one. The headline effect sizes were below Cohen’s small-effect benchmark and estimated at the limit of what this design could detect, and the cross-sectional data require longitudinal confirmation of the platform contrasts. Whether distinguishing platform types, rather than treating screen time as uniform, improves population-level digital well-being efforts remains a hypothesis for longitudinal and experimental testing.

## 1 Introduction

Whether time spent online carries a uniform mental health signature or one that depends on the platforms used by a population is a question of direct public health relevance. Depression and related psychological distress are among the leading contributors to the global burden of disease, and digital media saturate most adults’ daily routines. Even small platform-specific associations, when spread across a population, can accumulate to a non-trivial share of symptom burden. Yet the evidence needed to evaluate platforms at the population level remains thin, particularly outside high-income Western settings.

Debate over digital use and wellbeing has solidified into two competing perspectives. The first, articulated by Twenge (1) and Haidt (2), frames digital technology as a major driver of recent declines in adolescent and young adult mental health; the second, from Orben and Przybylski’s analysis of large nationally representative datasets (3) and Orben’s review of that literature (4), reports only small and variable associations. The gap is not merely rhetorical: measurement and analytic choices transform the same data into divergent substantive claims. Both perspectives converge on one premise—that problematic use is a behavioral pattern along a continuum rather than a binary clinical entity—consistent with Kardefelt–Winther’s view that excessive use reflects understandable coping rather than compulsive pathology (5). Clinical instruments such as the Smartphone Addiction Scale–Short Version (SAS-SV), the Bergen Social Media Addiction Scale (BSMAS), and the Mobile Phone Addiction Tendency Scale (MPATS) have sound measurement properties but are too burdensome for comprehensive national surveys, so prevalence-oriented studies have employed dedicated screeners (6, 7).

A behavioral indicator approach—using ordinary self-reported frequency and duration rather than a validated clinical scale—offers a practical route to the lower-intensity portion of the continuum. Parry and colleagues’ meta-analysis documents, across 66 effect sizes from 44 studies, a correlation of about 0.38 between self-reported and logged use (8). If this attenuation is broadly similar across platforms (an assumption the meta-analysis supports only indirectly: it found no significant difference between social media and phone measures, *r* = 0.35 versus 0.38, but did not test individual platforms), the relative ordering of platform coefficients is largely preserved under measurement error, so self-reported measures can still reveal systematic patterns at the population scale. Large self-report studies at that scale are nonetheless common, from 1,989 US adults (6) and 21,244 French adults (7) to Thomas and colleagues’ 30-country online panel survey of 15,000 internet users (9), although all three relied on screeners rather than ordinary use indicators.

Türkiye sits at the intersection of three coverage gaps: it falls outside the Arab Barometer that anchors the World Happiness Report 2026 Middle East and North Africa (MENA) chapter (10), is absent from the recent European Social Survey rounds on which the Report’s internet-use chapter draws, and receives only limited digital-use measurement in the Turkish World Values Survey. Existing Turkish research has largely focused on students or adolescents: Koç and colleagues identified three problematic-use profiles among 807 adults from 39 provinces, but a mean age of 23.35 years limits national inference (11), and Sarman and Tuncay documented platform-divergent mental-health correlates among Turkish adolescents (12). Platform heterogeneity—distinct mental-health signatures rather than one uniform screen-time effect—has thus been documented in Turkish adolescent and young adult samples but never tested against the broader adult population with a probability-based design.

The population that would carry such a test is now almost fully connected. According to the Turkish Statistical Institute’s 2024 Survey on ICT Usage in Households and by Individuals, 88.8% of those aged 16–74 used the internet, and the most widely used social media and messaging applications among individuals in that age range were WhatsApp, YouTube, and Instagram (86.2, 71.3, and 65.4%) (13), with daily social media use averaging 2 h and 44 min (14).

Two frameworks inform this population question, though only one guides the analysis. As background, the compensatory internet use model locates engagement in psychosocial difficulty rather than technology: digital use is a coping response to psychosocial problems such as loneliness and negative affect, which precede and motivate it (5). It explains why people turn to platforms but not why the experience deepens distress—a gap that platform structure and social comparison fill. A recent Chinese study extends this compensatory model to short-video addiction under stress (15), leaving that gap in place. The Differential Susceptibility to Media Effects model, which informs the analysis, posits that developmental, dispositional, and social-context moderators cause media effects to vary in size and even in direction across individuals and contexts; the present study operationalizes only its developmental (age) prediction (16). That developmental perspective fits an age gradient: younger users, whose identity buffers are plausibly less settled, are more exposed to upward social comparison and algorithmic content cycles than older adults. A meta-analysis of 141 active–passive studies found most pooled associations negligible (|*r*| < 0.10), with age as a key moderator (17), and a scoping review of the active–passive distinction (18) and Kross and colleagues’ review (19) likewise favor person- and content-specific susceptibility over a single coefficient.

That framework motivates treating social media as two architectures that differ in content selection, social comparison, and performative pressure: algorithm-driven content consumption (Instagram, TikTok, part of YouTube) and interpersonal communication (WhatsApp, iMessage, part of Telegram). Regional evidence is suggestive but incomplete: Bouazza and colleagues’ systematic review of 33 MENA studies (21,487 participants) linked problematic smartphone use to depression, anxiety, and stress and flagged application type as a determinant (20), while Al-Mohaimeed and colleagues found that using more than five applications, compared to three or fewer, doubled the odds of problematic use, with academic and religious uses being protective (21)—yet neither tested the algorithm-driven versus interpersonal contrast at the platform level. That contrast also reorganizes the loneliness question: displacement, in which digital use replaces face-to-face contact and raises loneliness (1), is expected on algorithm-driven platforms, whereas augmentation, in which contact strengthens ties (19), is expected on interpersonal ones. Generational evidence points to a boundary somewhere between the mid-30s and the mid-40s: Chapter 8 of the World Happiness Report 2026, using European Social Survey data and an instrumental-variable design, reports the internet–wellbeing relationship as strongly negative for Gen Z, moderately negative for Millennials, near zero for Gen X, and slightly positive for Baby Boomers. This cohort contrast is based on birth years rather than ages (Millennials 1981–1996, Gen X 1965–1980), which we convert to ages 43–44 in 2024, while its descriptive age curve turns from negative to positive at about 35 (22).

The present study addresses this population-level gap directly. Drawing on the 2024 wave of the Turkish General Social Survey, a probability-based national survey of 2,615 adults conducted by the İSAR Research Center, it tests whether platforms relate differently to psychological distress in the adult population and evaluates the claim that all screen time is equally harmful. The analysis advances five hypotheses.

*H1*. Daily social media use displays a small positive association with psychological distress after demographic adjustment.

*H2*. Age operates as a confounder of the association between digital use and distress for most platforms (so that adjustment partly absorbs the bivariate coefficient) and, in a component formed after inspecting the bivariate structure rather than in advance, as a suppressor for interpersonal messaging platforms such as WhatsApp (so that adjustment reveals a protective signal masked at the bivariate level); the suppression component is therefore an exploratory expectation.

*H3*. *Platform associations differ in direction*. *Algorithm-driven platforms* (Instagram in particular) predict elevated psychological distress; interpersonal communication platforms (WhatsApp in particular) predict reduced psychological distress.

*H4*. Age moderates the Instagram association. The Instagram association is stronger in younger respondents and approaches zero in older respondents.

*H5*. The 10-item CES-D-informed emotional-state battery fits a two-factor (negative and positive affect) solution in the Turkish adult sample—a measurement precondition for the composite outcome rather than a substantive association hypothesis.

An exploratory question accompanies these hypotheses: whether single-item loneliness (welalone) carries a detectable indirect pathway from digital use to distress (an exploratory mediation question). Because welalone is a single Likert item rather than a multi-item loneliness scale, such as the three-item loneliness scale of Hughes and colleagues (23), this test is underpowered relative to a full-scale measurement and is framed as exploratory rather than confirmatory.

## 2 Materials and methods

### 2.1 Data and sample

We analyzed the 2024 wave of the Turkish General Social Survey (Türkiye Genel Sosyal Saha Araştırması, TGSS) (24), a probability-based, nationally representative survey conducted by the İSAR Research Center (Abdullah Tivnikli İSAR Foundation, Istanbul) between 17 May and 2 June 2024. Interviewers approached respondents in person, and the questionnaire was then completed in one of two modes: interviewer-administered face-to-face interviewing (computer-assisted personal interviewing, CAPI; 1,614 respondents, 61.7%) or self-completion through a web link (computer-assisted self-interviewing, CASI; 1,001 respondents, 38.3%); 39.5% of the analytical sample completed the survey via the self-administered link. The public documentation does not state how the mode was assigned, and link completion was more common among younger and urban respondents (*r* with age = −0.23 in the analytical sample), so mode is treated as a covariate in a sensitivity check rather than as randomly assigned (Section 3.3). Modeled on the US General Social Survey, the European Social Survey, and the World Values Survey, and built on a sampling frame developed with the Turkish Statistical Institute (TÜİK), TGSS is a general-purpose omnibus survey rather than an instrument designed specifically for studying digital use and mental health. The present study makes secondary use of its digital-use, wellbeing, and demographic items, asking whether a platform-differentiated pattern in the population distribution of psychological distress can be identified from data gathered for broader descriptive purposes. The public-use dataset and codebook are openly available at https://analiz.tgss.org.tr.

The target population was the adult population of Türkiye, defined as all residents aged 18 and over, irrespective of citizenship, so that migrants, refugees, and foreign nationals were included. The sample was drawn using a probabilistic multistage stratified cluster design (24). The 12 Nomenclature of Territorial Units for Statistics level-1 (NUTS-1) regions formed the first-stage strata (described by the provider as primary sampling units), and the 26 provinces that give their names to the NUTS-2 subregions served as secondary units, with interviews allocated across them in proportion to provincial population (probability-proportional-to-size allocation). The TÜİK 2023 urban–rural classification then stratified locations into densely urban, semi-densely urban, and rural settlements, across which 134 clusters were drawn (80 densely urban, 27 semi-densely urban, 27 rural), each anchored to a TÜİK address block of roughly 500 households. Within each cluster, field workers approached every third eligible adult along the cluster route (an interval that was relaxed under team-leader guidance in low-footfall rural clusters), in public spaces (streets, squares, parks, and cafés) rather than in households; this fixed-interval rule limited interviewer discretion (24). Targeting a ±2% margin of error at 95% confidence and a minimum of 2,400 completed questionnaires, the field team extended invitations to 3,600 individuals and retained 2,615 valid cases after editing and quality control. Of these, 51 lacked a valid nine-item distress composite, 856 of the remainder lacked the total-internet-time item (853 of the 871 respondents assigned rotation form R1, who did not receive it by design, plus 3 item non-responses), and a further 230 lacked one or more of the other model variables (social media time 58, TikTok 61, Instagram 19, YouTube 12, WhatsApp 3, loneliness 6, household income 64, marital status 5, household size 2), leaving 1,478 complete cases for analysis.

TGSS uses a questionnaire-rotation system in which the instrument is split across seven item blocks distributed over three rotation forms (assignment patterns R1–R3): a core block of demographic, socioeconomic, and cultural items administered to all 2,615 respondents, three substantive blocks (with each respondent receiving two of the three), and three short supplements (with each respondent receiving one). Total internet time (onlinehour), which is included in an item block administered to roughly two-thirds of respondents, is the principal mechanism producing the gap between the full sample (*N* = 2,615) and the listwise-complete analytical sample (*N* = 1,478). This is because its 33.5% missingness in the full sample (875 of 2,615 respondents) far exceeds the 2–6% item-level missingness on the platform-frequency measures. Since rotation-form assignment is random by design, the rotation-block variables, chiefly total internet time, are missing completely at random (MCAR) with respect to the substantive variables. In contrast, item-level missingness on the distress composite and the loneliness item is not guaranteed to be MCAR and is treated as missing at random (MAR). The dominant component of form assignment therefore justifies the missing-at-random assumption underlying the multiple-imputation check. Listwise deletion across the dependent variable, the six demographic controls, the two exposure measures, the four platform-frequency indicators, and the loneliness item produced an analytical sample size of *N* = 1,478, whose demographic profile closely tracks that of the full sample concerning age, gender, education, and income (Supplementary Table S12).

### 2.2 Measures

#### 2.2.1 Psychological distress

The dependent variable was a nine-item past-week negative-affect (psychological-distress) index. The 2024 TGSS included a 10-item battery of past-week emotional-state items (variable prefix wel) with a four-point frequency format. Five items closely align with the 10-item Center for Epidemiologic Studies Depression Scale (CES-D-10) (25): depressed mood, restless sleep, everything an effort, felt lonely, and (reverse-scored) happy; a sixth item (could not get going) corresponds approximately. The remaining battery items draw on adjacent content that is not part of CES-D-10, notably an inability-to-stop-worrying item related to generalized-anxiety content (cf. the Generalized Anxiety Disorder-7 scale, GAD-7) and an inability-to-cope item related to perceived stress (cf. the Perceived Stress Scale, PSS). Four CES-D-10 items (bothered by things, trouble concentrating, hopeful about the future, and fearful) are absent. An item-by-item mapping is provided in Supplementary Table S11. Validated Turkish-language versions exist for the 20-item CES-D in an adult clinical sample (26) and for the adolescent CESD-R-10 (27), but this battery was not administered as the CES-D. The composite is therefore best understood as a CES-D-informed past-week negative-affect index rather than a validated CES-D administration (a population-level index, not a clinical or diagnostic instrument), and its structural validity was tested directly through the confirmatory factor analysis reported below. One item, “felt lonely” (welalone), is examined here as an exploratory covariate rather than a formal mediator; retaining it in the outcome would introduce a conceptual tautology, so a nine-item composite excluding welalone was constructed. This nine-item composite correlated at *r* = 0.992 with the full 10-item version.

The two positively worded items (welhappy, welenjoy) were reverse-coded as new value = 5-original value, given the 1-to-4 Likert anchors, ensuring that higher composite scores consistently indicate greater distress burden. The nine-item composite was computed as the mean of valid items multiplied by 9 to avoid downward bias under partial missingness, requiring at least seven of the nine items to be valid; within the analytical sample (*N* = 1,478), 1,385 respondents provided valid data on all nine items, 74 on eight, and 19 on seven. The composite scores ranged from 9 to 36 (*M* = 19.13, SD = 5.32), with skewness = 0.40 and kurtosis = 0.05, both falling within acceptable limits for parametric analysis. Internal consistency reached *α* = 0.883 for the full 10-item scale, *α* = 0.875 for the eight negatively worded items, *α* = 0.754 for the two positively worded items, and *α* = 0.875 for the nine-item composite used as the dependent variable.

#### 2.2.2 Digital use

Two families of digital-use measures were used. Two exposure measures—daily social media time (digsmhr) and total internet time (onlinehour)—used a 27-category ordinal scale (1 = “never”; 2 = “under 30 min”; 3 = “30–59 min”; then 1-h bands from 4 = “1 h” to 27 = “24 h”), with ordinal ranks treated as continuous and not directly interpreted as hours. Four platform-frequency measures captured use of TikTok, Instagram, YouTube, and WhatsApp on a seven-point scale (1 = never to 7 = several times a day); descriptive statistics are reported in the Results and Table 1. Three of these—WhatsApp, YouTube, and Instagram—are among the applications identified by TÜİK (13) as most widely used in Türkiye, while TikTok was included to capture short-video use despite its lower population-level penetration; Twitter and Facebook are analyzed separately in the latent profile analysis (see Supplementary material). Large-language-model use (digillm), which is included in an ancillary model reported in Supplementary Table S9, was measured on an eight-point frequency scale (1 = never to 8 = many times a day).

**Table 1:** Descriptive statistics for analytical sample variables (*N* = 1,478).

Variable	*N*	*M*/%	SD	Min	Max
Psychological distress (nine-item)	1,478	19.13	5.32	9	36
Age	1,478	39.53	14.88	18	84
Female (%)	1,478	48.4	–	0	1
Education	1,478	3.94	1.48	1	8
Household income	1,478	10.53	4.41	1	24
Never-married (%)	1,478	28.8	–	0	1
Household size (excl. respondent)	1,478	2.91	1.65	0	12
Social media time (daily)	1,478	4.64	2.69	1	27
Total internet time	1,478	5.91	3.34	1	27
TikTok frequency	1,478	2.45	2.32	1	7
Instagram frequency	1,478	5.03	2.54	1	7
YouTube frequency	1,478	4.82	2.29	1	7
WhatsApp frequency	1,478	6.25	1.72	1	7
Loneliness (welalone)	1,478	1.92	0.89	1	4

#### 2.2.3 Loneliness

A single item from the same emotional-state battery, welalone, rated on a four-point scale from 1 (rarely or never) to 4 (most or all of the time), served as an exploratory covariate (*M* = 1.92, SD = 0.89). Single-item measurement of loneliness is conventional in nationally representative surveys because of response burden, and although the nine-item outcome excludes welalone at the item level, both derive from the same past-week battery, so shared-method variance cannot be ruled out. Its association with psychological distress is estimated in Block 5 of the hierarchical regression, and its potential indirect role in the digital-use associations is examined through the exploratory bootstrap procedure described below.

#### 2.2.4 Demographic controls

Six controls entered the first regression block: age (continuous, 18–84), gender (female = 1), educational attainment (1–8 ordinal), household income (1–24 ordinal), a marital indicator, and household size, measured as the number of co-residents excluding the respondent (0 = lives alone) and treated as continuous. The marital indicator was derived from the six-category TGSS marital variable and coded 1 for never-married respondents (28.8% of the analytical sample) and 0 for all partnered or post-partnered statuses combined (currently married 61.5%, separated 2.6%, cohabiting 0.6%, divorced 4.2%, widowed 2.4%). Throughout, references to the “marital” coefficient denote this never-married-versus-all-other contrast. A further set of contextual variables—religiosity, region, lifetime unemployment experience, sleep, and maternal emotional closeness—entered ancillary models reported in the Supplementary material.

### 2.3 Statistical analysis

The analysis proceeded in five phases, followed by the identification of assumptions, diagnostics, and auxiliary procedures set out at the end of this section. First, the structural validity of the distress measure was examined through confirmatory factor analysis (CFA) on the complete 10-item battery, estimated on the 2,358 respondents with complete battery data, comparing a single-factor model with a two-factor (negative and positive affect) model, both estimated using the Python semopy package with maximum likelihood; each converged in fewer than fifty iterations. Fit was judged against the Hu and Bentler (28) cutoffs for good fit, with a comparative fit index (CFI) ≥ 0.95 and a root mean square error of approximation (RMSEA) ≤ 0.06, while the more lenient conventional thresholds of CFI ≥ 0.90 and RMSEA ≤ 0.08 were treated as acceptable; the Tucker–Lewis index (TLI) and the standardized root mean square residual (SRMR, for which Hu and Bentler suggest a cutoff close to 0.08) are reported for completeness. Model comparison rested on the fit indices (CFI, TLI, RMSEA) and the chi-square difference test rather than on information criteria, which the semopy implementation computes in a non- way. Configural invariance alone—the same factor structure across gender and the six age groups recorded in the survey (18–24, 25–34, 35–44, 45–54, 55–64, and 65 and over), without constraining loadings or intercepts—was assessed; full metric and scalar invariance await larger subgroup samples, and the configural evidence does not permit between-group latent mean comparisons.

Second, a Pearson product–moment correlation matrix among all analytical variables was computed listwise, with two-tailed significance derived from the standard *t*-transform on *N*-2 degrees of freedom.

Third, a five-block hierarchical ordinary least squares (OLS) regression with heteroskedasticity-consistent (HC3) robust standard errors was estimated. The continuous variables (age, digsmhr, onlinehour, and the four platform-frequency indicators) were mean-centered before entry and before constructing the age × platform interaction terms, reducing non-essential multicollinearity between the main effects and the interactions and giving the intercept a substantive meaning at the sample average. Standardized coefficients were computed as *β* = *B* × SD_x_/SD_y_, where *B* is the unstandardized OLS coefficient (HC3 affects only its standard error), with dummy-coded predictors (female, marital indicator) standardized in the same way. Block 1 included the six demographic controls; Block 2 added the two continuous exposure measures (social media time, total internet time); Block 3 added the four platform-frequency variables (TikTok, Instagram, YouTube, WhatsApp); Block 4 included five age × platform interactions (age × social media time and age × each platform; age × total internet time was omitted to keep the interaction set focused on the four platform-specific terms and the primary social media–time aggregate); and Block 5 added the single-item loneliness indicator (welalone) as an exploratory covariate.

Fourth, moderation in the Block-5 model was examined through a simple-slopes analysis of the Instagram association at three age levels (mean−1 SD, mean, mean+1 SD) and a Johnson–Neyman procedure tracing the Instagram association across the continuous age range; both procedures were also applied to the age × social media time interaction, with conditional slopes reported at ages 25, 40, 54, and 65.

Fifth, several sensitivity analyses were conducted, reported in Supplementary material or in Section 3.3. For the headline coefficients, NUTS-2 cluster-robust and wild-cluster-bootstrap standard errors were estimated, along with a re-fit on a five-item CES-D-mapped depressive subscale (the five non-loneliness items that map exactly or approximately onto CES-D-10; Supplementary Table S14). The simple-slopes and Johnson–Neyman procedures for the age × Instagram interaction were repeated on the Block-4 model, which omits the loneliness covariate (Supplementary Table S17). Models were also estimated separately within three age bands (18–29, 30–49, and 50 and over) without the loneliness covariate and without the age interaction terms (Supplementary Table S2). A mode-of-administration check added a self-completion indicator and its interactions with the three significant digital predictors to the headline model. Quantile regression was estimated at the 25th, 50th, 75th, and 90th percentiles (29), and a latent profile analysis covered seven digital-use indicators (social media time and TikTok, Instagram, YouTube, WhatsApp, Twitter, and Facebook frequency). The functional form of age was examined by adding a quadratic age term and its interaction with Instagram frequency to the full model. Further checks included an outcome restricted to the seven negatively worded items; winsorized, influence-trimmed, and log(1 + x)-transformed exposure scales; a re-estimation excluding total internet time, the predictor with the highest missingness; and checks for within-users and the daily-or-more dichotomy for WhatsApp (the dichotomy replacing the frequency term and its age interaction). A multiverse of 48 analytic paths spanning 24 distinct coefficient vectors (the HC1/HC3 choice affects standard errors, not point estimates) explored analytic flexibility across defensible modeling choices, and a missing-data comparison set evaluated listwise deletion against single mean imputation and multiple imputation by chained equations (*m* = 20) pooled under Rubin’s (30) rules with the Barnard–Rubin (31) degrees-of-freedom correction. Finally, a post-stratification raking reweighted the sample to the 2024 Türkiye adult population marginals via iterative proportional fitting (32) on age × gender and NUTS-1 cells from the Address-Based Population Registration System (ADNKS) release (33), using the exact 2024 adult population shares from the TÜİK single-age table (targets in the Supplementary Table S7 note). All *p*-values are two-tailed, and the conventional *α* = 0.05 governs significance decisions before the multiple-comparison correction described below.

#### 2.3.1 Identification assumptions

The regression coefficients can be interpreted causally only under explicit assumptions, some of which the design satisfies and some of which it does not (34, 35). The target estimand is the population-level association between platform-specific digital use and the nine-item distress composite, controlling for the demographic set. The principal backdoor pathways identified at the design stage—age, gender, education, household income, marital status, and household size—together reflect cohort, life-stage, and resource gradients that plausibly influence both digital adoption and distress prevalence, and Block 1 accounts for these at the standard demographic level. Loneliness, introduced in Block 5, is treated in the literature as both a potential confounder—in Kardefelt–Winther’s compensatory internet use framework, pre-existing loneliness drives compensatory digital use (loneliness → use), making it an antecedent common cause—and, under a different displacement account, as a potential mediator (digital use → loneliness → psychological distress). The cross-sectional design cannot determine which role loneliness plays. A third possibility is measurement rather than causal, since welalone is drawn from the same past-week battery as the outcome and loads on the same negative-affect factor (Section 3.1); its implications for the Block-5 estimate are discussed in Section 4.3. Any change in the Block-4 digital coefficients when loneliness is added in Block 5 is thus interpreted as a mediator-versus-confounder tension rather than as mediation, in line with Rohrer (34), with the exploratory bootstrap indirect-effect test reported in the Results. Several pathways remain open. Reverse causation cannot be ruled out, as already-distressed individuals may seek out Instagram-type platforms and disengage from WhatsApp-type interpersonal channels; unmeasured stable traits (personality, attachment style, prior trauma) may predispose respondents to both heavier platform use and higher symptom burden; measurement attenuation in self-reported digital use cannot be corrected using the present file (8); and residual within-cluster correlation at the address-block level cannot be modeled because the public-use file omits that identifier. The coefficients are therefore interpreted as conditional associations based on the demographic-adjustment set, with the loneliness-adjusted (Block-5) and partially-adjusted (Block-4) estimates reported separately.

#### 2.3.2 Diagnostics and corrections

HC3 robust standard errors were used throughout. Variance inflation factors (VIF) were computed for all 18 Block-5 predictors on the centered design matrix and ranged from 1.07 to 2.29, which is within the range considered unproblematic by Kutner et al. (36), so multicollinearity was not an issue. Because the analytic strategy encompasses a five-block regression, four contextual models, and two exploratory tests, the risk of false positives across the family of tests increases; therefore, the Benjamini–Hochberg false discovery rate (FDR) correction (37), which controls the expected proportion of false positives among rejected hypotheses, was applied to the twenty-test hypothesis family. These results are listed with raw and corrected *p*-values in Supplementary Table S16, with uncorrected *p*-values reported in the tables and text, and the FDR-adjusted values summarized in the robustness section. For tests with exact *p*-values below 0.001, the correction was applied to the conservative ceiling *p* = 0.001, yielding upper-bound adjusted values that do not change substantive conclusions.

#### 2.3.3 Common method bias

Because all psychological variables came from a single self-report instrument, common method bias was screened using Harman’s single-factor test (38), entering the eight self-report variables (the nine-item distress composite, the loneliness item, and the six digital-use measures) into an unrotated principal component analysis on the correlation matrix. The first component accounted for 33.9% of the variance (49.1% when the analysis was conducted at the item level across the 10 emotional-state items, *N* = 2,358 with complete battery data), which is below the conventional 50% threshold. This result should be interpreted as the absence of an overwhelming common factor rather than as positive evidence that common method variance is absent. Because the platform-frequency predictors are behavioral items outside the emotional-state battery, the platform–distress associations do not share item-level content with the outcome. Harman’s test is a weak indicator by contemporary standards (38), and simulation work shows that common method variance must be present at substantively high levels to meaningfully bias relationships at typical reliabilities (39). Stronger procedures were not applicable: the marker-variable approach (40) requires a theoretically unrelated marker item that the TGSS 2024 instrument does not contain, and the unmeasured-latent-method-construct CFA (41), which needs no marker, requires multiple indicators per construct, whereas all of the self-report measures here, except the distress composite, are single items.

#### 2.3.4 Exploratory indirect-effect test, effect sizes, and power

The exploratory indirect pathway through loneliness was evaluated using a percentile bootstrap with 5,000 resamples. For each of the three digital predictors with significant main effects (social media time, Instagram, WhatsApp), the indirect effect was calculated as the product of a path (predictor → loneliness, adjusting for the six demographic covariates) and the b path (loneliness → psychological distress, adjusting for the predictor, all covariates, and the age × Instagram term). The 2.5th and 97.5th percentiles of the bootstrap distribution formed the 95% confidence interval, and an indirect effect was deemed significant when this interval excluded zero. Standardized coefficients provided the primary effect-size metric, interpreted against Cohen’s (42) benchmarks (small ≥ |0.10|, medium ≥ |0.30|, large ≥ |0.50|; applied to standardized coefficients only as a rough heuristic) and supplemented by Δ*R*^2^. Differences between two platform coefficients were tested directly on the standardized scale, with the variance of the difference obtained from the HC3 covariance of the two coefficients and referred to the standard normal distribution. The difference between the Block-4 and Block-5 age × Instagram coefficients was assessed using a percentile bootstrap of 2,000 resamples, refitting both blocks within each resample and taking the 2.5th and 97.5th percentiles of the standardized difference. Coefficients below the small-effect threshold are reported as statistically significant where *p* < 0.05 but interpreted cautiously, with their measurement-attenuation context discussed in Section 4.1. Because post-hoc power yields inconsistent inferences (43), we instead report the minimum detectable effect as a description of design sensitivity rather than as evidence for null effects (for which the coefficient confidence intervals in the Results are the relevant quantity). With *N* = 1,478, *α* = 0.05, and 18 predictors including the five interaction terms, the minimum detectable coefficient at 80% power was approximately *β* = 0.06–0.07 (Cohen’s *f*^2^ ≈ 0.005–0.008 under *f*^2^ = *β*^2^/(1 − *R*^2^) with the Block-5 *R*^2^ of 0.359; approximate, since *β*^2^ equals the semi-partial *R*^2^ only under orthogonal predictors), with a corresponding minimum detectable Δ*R*^2^ ≈ 0.003–0.005. The empirical HC3 standard errors imply minimum detectable standardized coefficients of 0.073 for Instagram, 0.076 for WhatsApp, 0.078 for the age × Instagram term, 0.085 for the age × social media time term, and 0.102 for social media time (Supplementary Table S13); these coefficient-specific values supersede the pooled analytic figure above. Each headline coefficient falls below its own threshold, which for a two-tailed 0.05 test at 80% power equals 2.80 standard errors, and is therefore equivalent to being significant at 0.05 but not at 0.005, so the comparison restates the *p*-values rather than adding evidence to them.

#### 2.3.5 Sampling weights

The public-use file (664 variables, 2,615 respondents) contains no survey design weight and no address-block cluster identifier; the released design identifiers include the 12 NUTS-1 first-stage strata, the 26 NUTS-2 subregions, and the three-level settlement-type stratum, consistent with anonymity-protection conventions. The provider describes the design as equal-probability (allocation proportional to population, with systematic selection of every third eligible adult) and does not publish any weights (24); because the address-block identifier is also withheld, cluster-robust standard errors at that level cannot be computed, and we instead report NUTS-2 cluster-robust standard errors (Supplementary Table S13) alongside the HC3 main specification. Compared with the 2024 population register (33), the realized sample matches the adult population on gender (women 49.1% in the full sample versus 50.4% in the register) and region (all 12 NUTS-1 shares within 3 points) but under-represents older adults (aged 65 and over: 7.8% versus 14.3%; women aged 50 and over: 9.9% versus 19.1%), as expected from recruitment in public spaces. For the association estimates reported here (the study estimates no population prevalences), unweighted regression is consistent when the model conditions on the covariates that govern selection, and weighting then incurs an efficiency cost if the errors are homoskedastic (44). Because the departures from the population lie on observed covariates that enter every model (age, gender), this condition is plausibly met; region, by contrast, enters only the contextual NUTS-1 model (Supplementary Table S8), the NUTS-2 clustered standard errors, and the raking sensitivity, so robustness to the full set of population margins is examined empirically below. Under the ADNKS raking sensitivity (Supplementary Table S7), the weighting (Kish) design effect of the raking (1.21, effective *N* ≈ 1,223) and the stability of the headline coefficients under reweighting indicate that the unweighted HC3 main specification is not seriously biased by the absence of design weights, though residual within-cluster correlation cannot be ruled out.

## 3 Results

### 3.1 Sample and measurement

The analytical sample comprised 1,478 adults drawn from a nationally representative probability survey. Respondents averaged 39.53 years of age (SD = 14.88, range 18–84); 48.4% were women, 28.8% were never married, and respondents reported an average of 2.91 co-residents (excluding themselves; 0 = living alone). Mean educational attainment scored 3.94, and mean household income was 10.53 on their respective ordinal scales. The nine-item distress composite averaged 19.13 (SD = 5.32, range 9–36) with a mild right skew (skewness 0.40, kurtosis 0.05), and its distribution showed the discreteness expected of a nine-item ordinal composite (Figure 1). Self-reported digital-use intensity was highest among the youngest respondents and declined across successive age groups (Figure 2).

**Figure 1:**
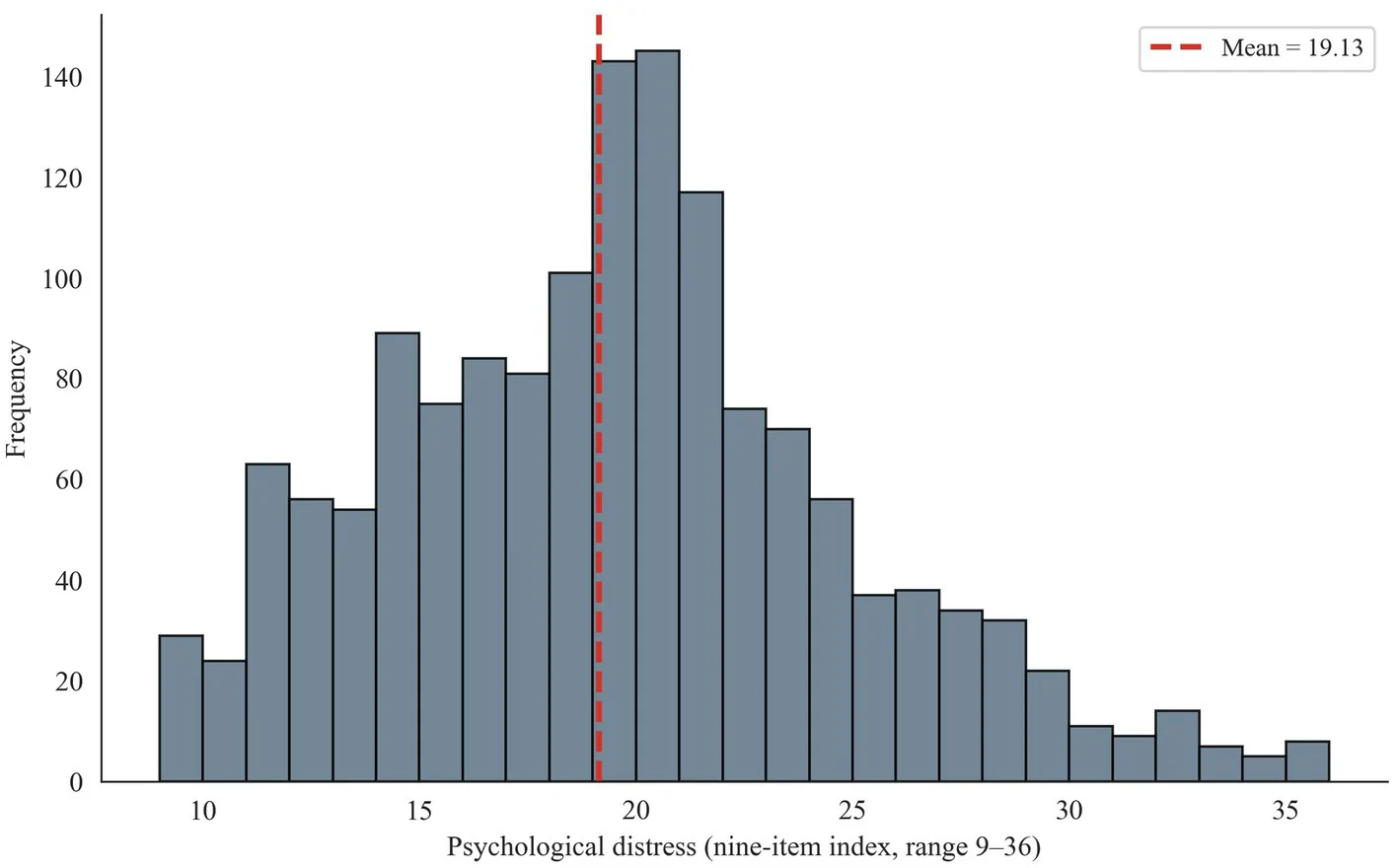
Distribution of the nine-item distress composite in the analytical sample (*N* = 1,478).

**Figure 2:**
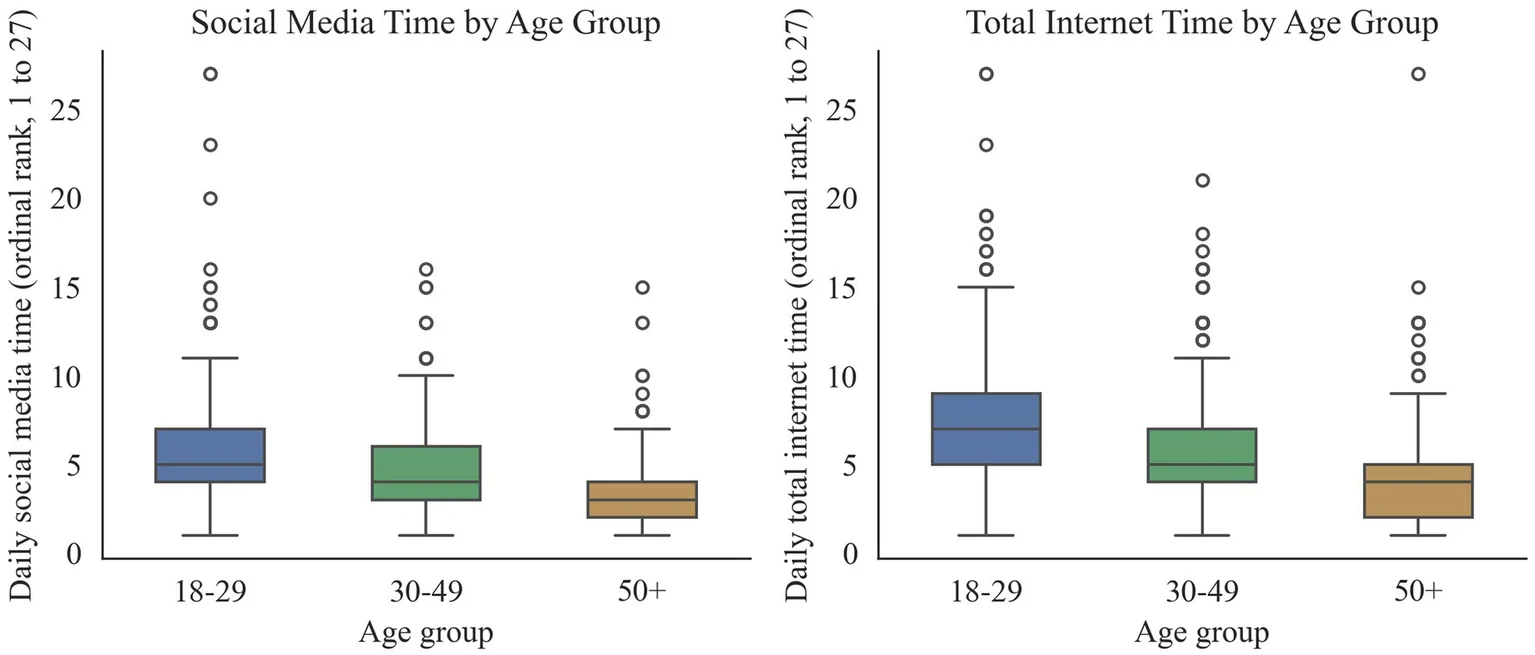
Distributions of digital-use intensity by age group (analytical sample, *N* = 1,478). The vertical axis in both panels is a 27-category ordinal rank (rank 4 = 1 h, 5 = 2 h, and so on to 27 = 24 h), not a direct hour count.

Digital-use behaviors were distributed unevenly across the population. Self-reported daily social media time averaged 4.64 (SD = 2.69, range 1–27), a rank falling between the 1-h and 2-h bands, and total daily internet time averaged 5.91 on the same 27-category ordinal scale. Platform-frequency means followed a clear hierarchy: WhatsApp was highest (*M* = 6.25, SD = 1.72), falling between the “once a day” and “several times a day” levels and approaching the scale ceiling; because use is near universal, the restricted variance means the protective association reported below disproportionately reflects the minority of lighter users, who may differ systematically, warranting added caution (a within-users check is reported in Section 3.3). Instagram (*M* = 5.03) and YouTube (*M* = 4.82) corresponded to use “a few times a week,” while TikTok (*M* = 2.45; 69.0% never used it) fell between “once a month or less” and “a few times a month” (Table 1).

The structural validity of the distress instrument was examined by comparing two competing confirmatory models (Supplementary Table S1). The single-factor model returned *χ*^2^(35) = 1,051.25, *p* < 0.001, CFI = 0.894, TLI = 0.864, RMSEA = 0.111, and SRMR = 0.055; with CFI below 0.90 and RMSEA above 0.10, the single-factor structure was not adequate in the Turkish adult sample. The two-factor model, in which the eight negatively worded items loaded onto a negative-affect factor and the two positively worded items onto a positive-affect factor (the two items entered in reverse-coded form), met conventional criteria: *χ*^2^(34) = 516.19, *p* < 0.001, CFI = 0.950, TLI = 0.934, RMSEA = 0.078, SRMR = 0.035. The two-factor model fit significantly better than the single-factor model based on the chi-square difference test (Δ*χ*^2^(1) = 535.06, *p* < 0.001), and the global fit indices clearly favored the two-factor solution.

All standardized loadings were significant (*p* < 0.001) and fell below unity. On the negative-affect factor, they ranged from 0.57 (welsleep) to 0.74 (welsad and welworry), with weldepp at 0.72, welhard at 0.71, welover at 0.72, welstop at 0.68, and welalone at 0.61. On the positive-affect factor, welhappy loaded at 0.76 and welenjoy at 0.79. The two latent factors correlated at *r* = 0.66, and all error variances were positive. The two-factor configuration was recovered in both gender groups (male CFI = 0.958, RMSEA = 0.071; female CFI = 0.938, RMSEA = 0.083) and in all six age groups (CFI ranging from 0.935 to 0.962). However, RMSEA exceeded the 0.08 ceiling adopted here in the female group and in four of the six age groups (0.082–0.090; the remaining two groups were 0.072 and 0.075), so configural invariance is supported only partially across both groupings. Because metric invariance could not be tested at these subgroup sizes, the age moderation reported below rests on the assumption that the composite carries the same measurement structure across age, and that assumption is supported here only in part. Internal consistency was high: Cronbach’s alpha reached 0.883 for the full 10-item scale, 0.875 for the eight-item negative-affect subscale, 0.754 for the two-item positive-affect subscale, and 0.875 for the nine-item composite used as the outcome.

### 3.2 Platform-specific associations and age moderation

The bivariate structure (Table 2) indicated that screen time carries no single, uniform risk signature at the population level. Among individual predictors, loneliness had the strongest positive correlation with psychological distress (*r* = 0.545), followed by female gender (*r* = 0.199) and the never-married indicator (*r* = 0.130), while education (*r* = −0.081) and household income (*r* = −0.185) correlated negatively. The negative age–distress correlation (*r* = −0.209) shows that younger adults bore the higher distress burden. Digital use, in turn, was patterned by age: social media time (*r* = −0.391), total internet time (*r* = −0.386), Instagram (*r* = −0.398), YouTube (*r* = −0.381), TikTok (*r* = −0.256), and WhatsApp (*r* = −0.272) all decreased as age increased. The raw associations of digital use with psychological distress were small: social media time *r* = 0.109, Instagram *r* = 0.102, TikTok *r* = 0.099, YouTube *r* = 0.039 (ns), and WhatsApp *r* = −0.028 (ns).

**Table 2:** Pearson correlations of variables with psychological distress and age (*N* = 1,478).

Variable	Psychological distress (*r*)	Age (*r*)
Loneliness	0.545^***^	−0.133^***^
Age	−0.209^***^	1.000
Female	0.199^***^	−0.139^***^
Education	−0.081^**^	−0.218^***^
Household income	−0.185^***^	−0.102^***^
Never-married	0.130^***^	−0.568^***^
Household size	0.077^**^	−0.307^***^
Social media time	0.109^***^	−0.391^***^
Total internet time	0.053^*^	−0.386^***^
TikTok frequency	0.099^***^	−0.256^***^
Instagram frequency	0.102^***^	−0.398^***^
YouTube frequency	0.039	−0.381^***^
WhatsApp frequency	−0.028	−0.272^***^

^*^p < 0.05. ^**^p < 0.01. ^***^p < 0.001. All correlations computed on the nine-item distress composite (mean of valid items × 9) using listwise deletion within the analytical sample.

A five-block hierarchical regression with HC3 robust standard errors partitioned these associations (Table 3). Block 1, which included demographic controls alone, reached *R*^2^ = 0.111; age was the strongest predictor (*β* = −0.175, *p* < 0.001), with female gender (*β* = +0.145, *p* < 0.001), household income (*β* = −0.165, *p* < 0.001), and the never-married indicator (*β* = +0.080, *p* = 0.010) also contributing—consistent with the epidemiological pattern of somewhat higher distress levels among never-married adults. Block 2 added the two time measures (Δ*R*^2^ = 0.004): social media time showed a small positive association (*β* = +0.068, *p* = 0.029), whereas total internet time did not (*β* = −0.004). Total internet use aggregates news, work, shopping, and education, a composition that the survey does not measure, which plausibly dilutes the population-level signal isolated by social media time. Block 3 added the four platform frequencies (Δ*R*^2^ = 0.008). The aggregate social media time coefficient in this block fell just short of significance (*p* = 0.051), suggesting that platform composition carries the signal. This block provided direct support for platform heterogeneity: Instagram had a positive coefficient (*β* = +0.074, *p* = 0.013), and WhatsApp had a protective one (*β* = −0.081, *p* = 0.008), while TikTok and YouTube remained null. Block 4 added five age × platform interactions (Δ*R*^2^ = 0.005), with age × Instagram at the threshold of significance (*β* = −0.060, *p* = 0.059).

**Table 3:** Hierarchical regression results (dependent variable: Nine-item distress composite computed as mean × 9, *N* = 1,478, HC3 robust standard errors).

Variable	Block 1	Block 2	Block 3	Block 4	Block 5	Block 5 95% CI
Age	−0.175^***^	−0.153^***^	−0.142^***^	−0.147^***^	−0.110^***^	[−0.174, −0.046]
Female	+0.145^***^	+0.144^***^	+0.144^***^	+0.149^***^	+0.113^***^	[0.068, 0.158]
Education	−0.058^*^	−0.061^*^	−0.056	−0.052	−0.039	[−0.089, 0.010]
Household income	−0.165^***^	−0.172^***^	−0.168^***^	−0.168^***^	−0.090^***^	[−0.140, −0.041]
Never-married	+0.080^**^	+0.077^*^	+0.065^*^	+0.058	+0.003	[−0.053, 0.059]
Household size	−0.009	−0.006	+0.001	+0.007	+0.014	[−0.030, 0.057]
Social media time	–	+0.068^*^	+0.063	+0.078^*^	+0.088^*^	[0.017, 0.159]
Total internet time	–	−0.004	−0.004	−0.001	−0.001	[−0.067, 0.065]
TikTok	–	–	+0.031	+0.044	+0.018	[−0.029, 0.064]
Instagram	–	–	+0.074^*^	+0.079^**^	+0.066^*^	[0.015, 0.117]
YouTube	–	–	+0.003	+0.003	+0.014	[−0.041, 0.068]
WhatsApp	–	–	−0.081^**^	−0.074^*^	−0.073^**^	[−0.126, −0.020]
Age × Social media time	–	–	–	+0.039	+0.070^*^	[0.011, 0.130]
Age × Instagram	–	–	–	−0.060	−0.069^*^	[−0.124, −0.014]
Age × TikTok	–	–	–	+0.038	+0.030	[−0.014, 0.074]
Age × YouTube	–	–	–	−0.028	−0.030	[−0.086, 0.025]
Age × WhatsApp	–	–	–	−0.011	−0.022	[−0.083, 0.039]
Loneliness	–	–	–	–	+0.498^***^	[0.446, 0.550]
*R* ^2^	0.111	0.114	0.122	0.127	0.359	
Adjusted *R*^2^	0.107	0.109	0.115	0.116	0.351	
Δ*R*^2^	0.111	0.004	0.008	0.005	0.232	

^*^p < 0.05. ^**^p < 0.01. ^***^p < 0.001. The final column, headed Block 5 95% CI, reports the 95% confidence interval of the Block-5 standardized coefficient and not that of any earlier block. An interval excluding zero corresponds to *p* < 0.05.

Block 5 entered the single loneliness item as an exploratory covariate (Δ*R*^2^ = 0.232). Loneliness was the strongest single predictor (*β* = +0.498, *p* < 0.001) and increased model fit to *R*^2^ = 0.359, an increment that reflects shared content and method variance with the outcome rather than a distinct explanatory contribution. Its inclusion shifted the digital coefficients in both directions: social media time strengthened from *β* = +0.078 to *β* = +0.088 (*p* = 0.016), Instagram declined from *β* = +0.079 to *β* = +0.066 but remained significant (*p* = 0.011), WhatsApp held at *β* = −0.073 (*p* = 0.007) with its protective direction intact, and the age × Instagram interaction reached significance at *β* = −0.069 (*p* = 0.014). In Block 4, before loneliness was entered, this interaction was non-significant (*β* = −0.060, *p* = 0.059), indicating that its significance is specification-dependent given loneliness’s unresolved mediator-versus-confounder role. The conditional slopes and the Johnson–Neyman bounds are therefore reported for both blocks (Supplementary Table S17), and the loneliness-free age-stratified slopes (Supplementary Table S2) remain broadly consistent. The Instagram and WhatsApp coefficients differ significantly from one another in the full model (standardized difference = 0.139, *z* = 3.44, *p* < 0.001), representing a directly tested contrast rather than one inferred from separate significance, whereas Instagram did not differ significantly from TikTok (*p* = 0.184) or YouTube (*p* = 0.180). An age × social media time interaction also emerged (*β* = +0.070, *p* = 0.020), indicating that Instagram is the operative indicator among younger adults, while overall social media time takes precedence among older adults. Like the age × Instagram term, it was non-significant before loneliness was entered (Block 4 *β* = +0.039, *p* = 0.247) and shares the same specification dependence. It is also the coefficient the wild-cluster bootstrap leaves short of 0.05 (*p* = 0.067; Section 3.3), although the age-stratified models reproduce the same shift without loneliness or interaction terms (Supplementary Table S2). Probing it showed the inverse pattern of the Instagram gradient, with the social media time slope negligible at age 25 (*β* = +0.020, *p* = 0.499) and rising to *β* = +0.090 at 40 (*p* = 0.015), +0.155 at 54 (*p* = 0.008), and +0.206 at 65 (*p* = 0.009), significant from roughly 34 years onward (Johnson–Neyman). None of the other age × platform interactions approached significance (TikTok *β* = +0.030, *p* = 0.183; YouTube *β* = −0.030, *p* = 0.285; WhatsApp *β* = −0.022, *p* = 0.481), so the age moderation is confined to Instagram and overall social media time. Within the demographic block, the age coefficient fell from *β* = −0.147 to *β* = −0.110 and household income from *β* = −0.168 to *β* = −0.090 (both still significant), whereas the never-married indicator was completely absorbed (*β* = +0.003, *p* = 0.920; see Section 4.3). Figure 3 summarizes the six Block-5 digital-use coefficients, with Instagram and social media time on the risk side of zero, WhatsApp on the protective side, and total internet time, TikTok, and YouTube clustered at zero.

**Figure 3:**
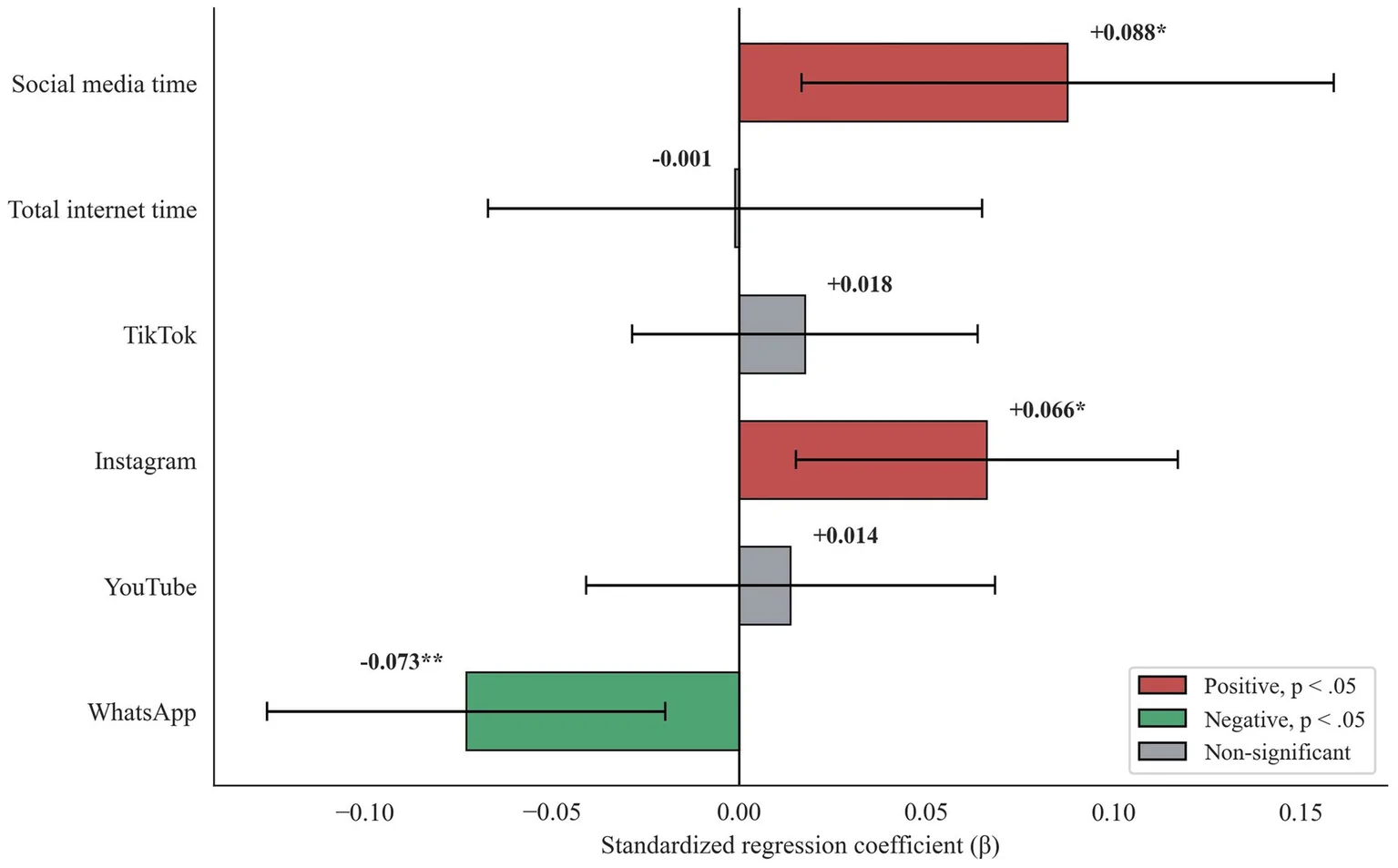
Standardized coefficients of the six digital-use measures (two exposure measures and four platform frequencies) in the full model (*N* = 1,478). Red indicates positive and significant at *p* < 0.05; green indicates negative and significant at *p* < 0.05; Gray indicates non-significant.

An exploratory bootstrap test qualified the role of loneliness. A percentile bootstrap (5,000 resamples) estimated the indirect effect of each of the three digital predictors with significant main effects (social media time, Instagram, WhatsApp) through loneliness, with all covariates and the age × Instagram term held constant. None of the intervals excluded zero: Instagram *β* = +0.014, 95% CI [−0.013, 0.042]; social media time *β* = +0.010, 95% CI [−0.018, 0.039]; WhatsApp *β* = +0.003, 95% CI [−0.026, 0.030]. Loneliness therefore functions as a strong independent correlate of psychological distress, with no detectable indirect pathway through the single-item measure; given that measure’s limited power, a small mediated component cannot be ruled out.

Because the age × Instagram interaction was significant in the loneliness-adjusted full model, simple slopes were estimated from that model at three age levels with age centered (mean = 39.53, SD = 14.88). The unstandardized Instagram slope was *B* = +0.277 (*t* = 3.45, *p* < 0.001) at the young level (24.7 years, *M*−1 SD), *B* = +0.139 (*t* = 2.54, *p* = 0.011) at the mean (39.53 years), and *B* = +0.001 (*t* = 0.01, *p* = 0.992) at the older level (54.4 years, *M* + 1 SD); the standardized slopes were *β* = +0.132, +0.066, and +0.000 (Table 4; Figure 4). At the young level, a one-unit rise in Instagram frequency corresponds to roughly 0.28 points on the 9-to-36 composite, halving to *B* = +0.139 at the mean and vanishing by the older level. Thus, the platform’s predictive capacity declines approximately linearly with age, a pattern predicted by the developmental reading of the Differential Susceptibility to Media Effects model, although a cohort or adoption gradient could produce the same pattern in a single cross-section (Section 4.4).

**Table 4:** Instagram simple slopes at three age levels (Block 5 model, nine-item dependent variable).

Age level	Approximate age	Instagram slope (unstandardized)	SE	*t*	*p*	Standardized slope (*β*)
Young (*M*−1 SD)	25	+0.277	0.080	3.45	<0.001	+0.132
Mean (*M*)	40	+0.139	0.055	2.54	0.011	+0.066
Older (*M* + 1 SD)	54	+0.001	0.076	0.01	0.992	+0.000

**Figure 4:**
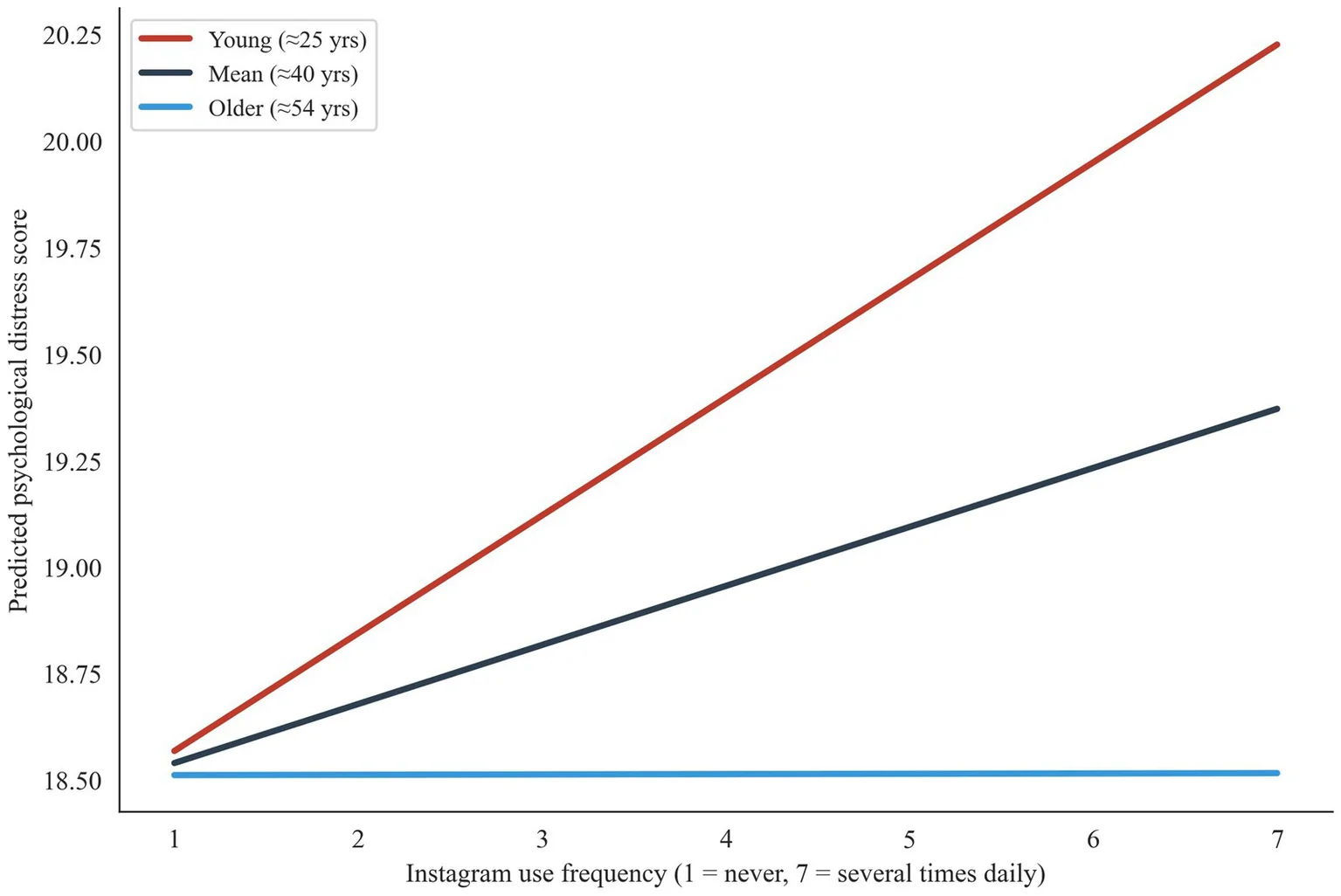
Instagram-by-age interaction (simple slopes) from the Block 5 model, which adjusts for loneliness. Supplementary Table S17 reports the corresponding slopes from the Block 4 model, where loneliness is not included.

The Johnson–Neyman procedure traced this decline across the continuous age range. The Instagram association remained significantly positive from age 18 into the early forties before losing significance, with the slope beginning near *B* = +0.34 at age 18 and its confidence band crossing zero at approximately 42–43 years (Figure 5). Both procedures were estimated on the Block-5 model. Repeated on the Block-4 model, in which loneliness is not entered, the conditional slope followed the same descending path, from *B* = +0.339 at age 18 to *B* = +0.047 at 54, and remained significant from 18 to approximately 45 years (Supplementary Table S17). The descending profile is therefore present in both specifications, while the interaction term itself clears the conventional threshold only once loneliness is entered. Because all continuous predictors are mean-centered, each block’s Instagram coefficient is the conditional slope at the mean age, and that slope is significant in Block 4 (*p* = 0.008); a region of significance therefore had to exist there, and its extent is not independent evidence for the moderation.

**Figure 5:**
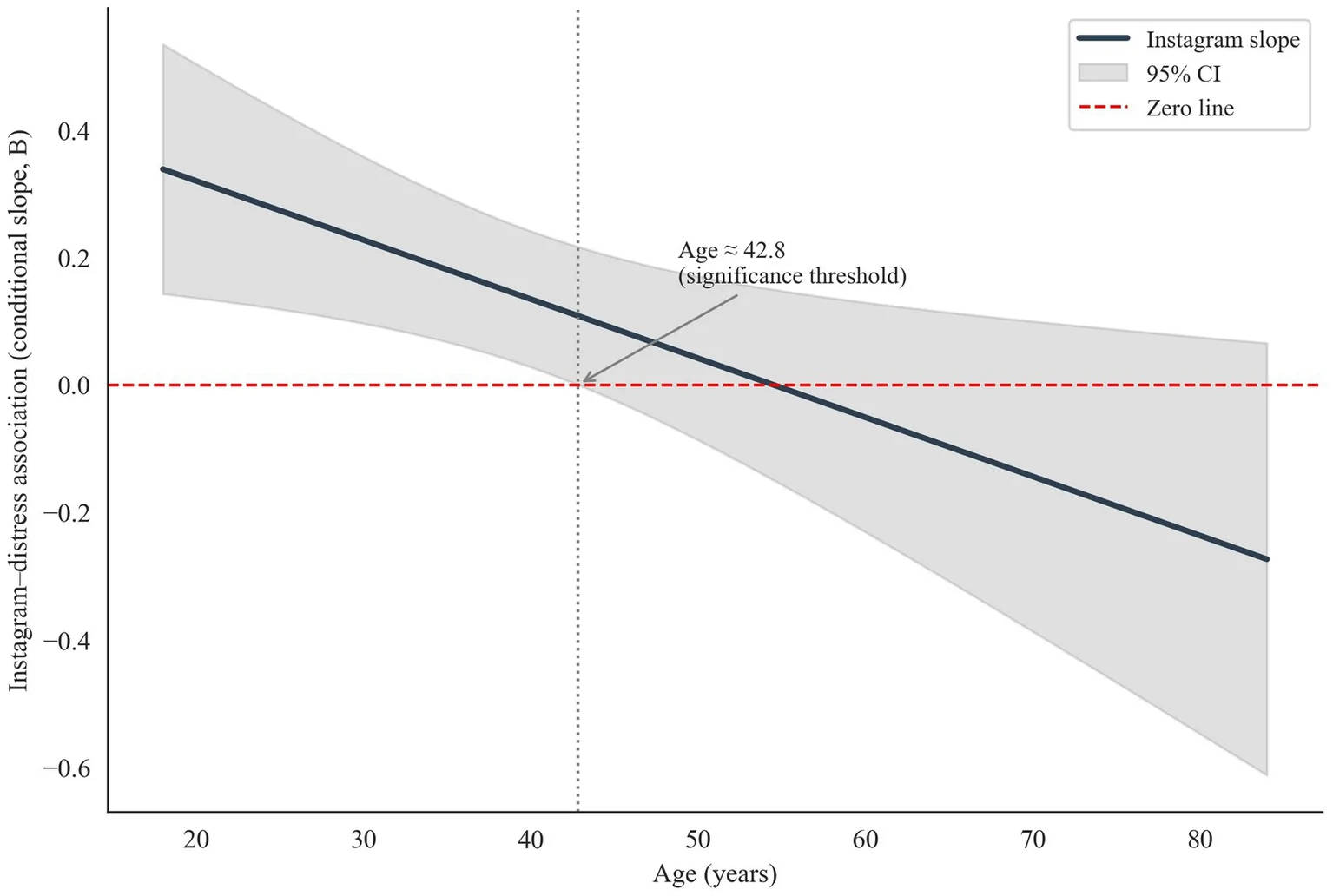
Instagram association across age (Johnson–Neyman) from the Block 5 model, which adjusts for loneliness. The shaded area is the 95% confidence interval. Supplementary Table S17 reports the corresponding Johnson–Neyman bounds from the Block 4 model.

Stratified models across three substantively defined age bands were broadly consistent with the continuous interaction (Supplementary Table S2). The bands (18–29, 30–49, and 50 years and older) encompass the empirical Johnson–Neyman point. In the 18–29 group (*N* = 451), no platform crossed the 0.05 threshold (Instagram *β* = +0.091, *p* = 0.081); coefficients of this size fall below what the smaller subsample can reliably detect, and the positive Instagram estimate is consistent in direction with, rather than evidence against, the continuous interaction. The band estimates are not monotonically ordered, as the 30–49 coefficient exceeds that of the 18–29 group, indicating that these models highlight the contrast between the younger bands and the oldest rather than the shape of the gradient across them. In the 30–49 group (*N* = 649, *R*^2^ = 0.125), platform associations were most clearly differentiated: Instagram carried the strongest positive coefficient (*β* = +0.112, *p* = 0.008) and WhatsApp a protective one (*β* = −0.098, *p* = 0.010). In the 50 + group (*N* = 378, *R*^2^ = 0.121), the situation shifted, with daily social media time now dominant (*β* = +0.173, *p* = 0.010) and WhatsApp non-significant (*β* = −0.131, *p* = 0.053), while neither algorithm-driven platform reached significance; the WhatsApp association was thus significant only in the 30–49 band, null among 18- to 29-year-olds (*β* = −0.003), and borderline among those aged 50 and over. The explanatory power of the model persisted at older ages even as the focus shifted from Instagram to overall social media time.

### 3.3 Robustness

The platform contrasts held across most sensitivity checks (multiverse summarized below), with the exceptions noted here. Cluster-robust standard errors computed at the NUTS-2 level left all five headline digital coefficients significant (Supplementary Table S13). Because the 26 NUTS-2 subregions fall below the large-sample threshold at which cluster-robust standard errors are reliable, a restricted wild-cluster bootstrap (Rademacher weights, 9,999 replications) provided a more demanding few-cluster test (Supplementary Table S15): the two platform contrasts and the age × Instagram interaction remained significant (Instagram *p* = 0.036, WhatsApp *p* = 0.024, age × Instagram *p* = 0.044), whereas the aggregate social- media time coefficient (*p* = 0.063) and the age × social media time interaction (*p* = 0.067) no longer met the 0.05 threshold, marking the platform contrasts as the most robust elements of the pattern. The platform associations also held on a five-item CES-D-mapped depressive subscale that overlaps substantially with the composite (*r* = 0.95), so this serves as an internal-consistency check rather than independent replication, though the age × Instagram moderation was attenuated there as well (*β* = −0.049, *p* = 0.100; Supplementary Table S14). Restricting the outcome to the seven negatively worded items (excluding loneliness; *N* = 1,462) left the platform and aggregate coefficients and the age × Instagram interaction intact (social media time *β* = +0.082, *p* = 0.023; Instagram *β* = +0.069, *p* = 0.008; WhatsApp *β* = −0.096, *p* < 0.001; age × Instagram *β* = −0.072, *p* = 0.013), with only the age × social media time interaction attenuating (*β* = +0.054, *p* = 0.077), indicating that the two positively worded items do not drive the platform pattern. Winsorizing the two 27-category exposure scales at their 99th percentile, dropping 86 high-influence cases (Cook’s D > 4/*N*), or log-transforming the ranks left the platform coefficients and the age × Instagram interaction essentially unchanged (Instagram *β* = +0.066 to +0.075, WhatsApp *β* = −0.072 to −0.073, age × Instagram *β* = −0.065 to −0.069, all *p* < 0.02), whereas the two aggregate social media time terms were sensitive to these choices (main effect *β* = +0.054 to +0.075, *p* = 0.005 under influence exclusion, 0.033 under winsorizing, and 0.109 under the log transform; age × social media time *p* < 0.001, 0.034, and 0.136, respectively), consistent with their fragility under the wild-cluster bootstrap. The WhatsApp association does not rely on respondents who never use the application: it remained negative among users only (*N* = 1,361, excluding the 117 who reported never using it; *β* = −0.059, *p* = 0.012), where the contrast is carried by the 99 respondents who use the application but less than daily, against the 1,262 reporting daily or more, and it held when use was dichotomized at daily or more versus less (85.4% daily or more; *B* = −1.02, *p* = 0.006). Quantile regression identified the social media time association in the high-distress tail (Q90 *β* = +0.128, *p* = 0.022) and the protective-direction WhatsApp coefficient at the median (Q50 *β* = −0.123, *p* < 0.001), while the Instagram association appeared across the lower and upper percentiles (Supplementary Table S3). A latent profile analysis of seven digital-use indicators (estimated on the broader *N* = 2,311 sample, listwise on the seven indicators together with the dependent measure, age, and gender) resolved onto an intensity axis that did not differentiate psychological distress (one-way analysis of variance, ANOVA, *F* = 1.07, *p* = 0.342), underscoring that frequency and content must be modeled jointly rather than as a single use-intensity gradient (Supplementary Tables S4, S5). Two missing-data analyses reproduced the direction of the results: mean imputation attenuated the Instagram and WhatsApp magnitudes by about a third (Supplementary Table S6), and multiple imputation by chained equations (m = 20, *N* = 2,564, pooled *R*^2^ = 0.374) attenuated them by a comparable margin (Supplementary Table S7). Excluding total internet time, the predictor with the highest missingness (33.5%), raised the analytical sample to 2,255 and preserved every headline coefficient in direction and significance (social media time *β* = +0.073, Instagram *β* = +0.049, WhatsApp *β* = −0.042, age × Instagram *β* = −0.059, age × social media time *β* = +0.050, all *p* < 0.05). Because these effects are small, their magnitudes varied across defensible methods; the sensitivity program is therefore best read as demonstrating stable direction with method-dependent magnitude rather than uniform robustness of effect size. A raking reweight to the exact 2024 ADNKS population margins (age × gender and NUTS-1 region; effective *N* ≈ 1,223; Supplementary Table S7) enlarged rather than attenuated several platform coefficients, and a 48-path multiverse analysis addressing the analytic-flexibility critique (45) held the direction of all four coefficients entered in the multiverse across every defensible path, with conventional significance retained in 97.9% (Instagram), 89.6% (social media time), 87.5% (WhatsApp), and 77.1% (age × Instagram) of paths; the multiverse fitted the two focal interactions (age × Instagram and age × social media time) rather than the full five-term interaction set. Four contextual models (religiosity, region, lifetime unemployment experience and sleep, and maternal closeness) are reported in Supplementary Table S8. The Instagram and WhatsApp coefficients were preserved in the religiosity and region models (*N* = 2,178 and 2,305; larger because these models omit total internet time and the age × platform terms), whereas in the unemployment-and-sleep model, fitted on a different rotation subsample (*N* = 1,463, of whom 688 belong to the analytical sample), both were non-significant before (*p* = 0.209 and 0.348) as well as after (*p* = 0.275 and 0.248) the added variables entered, so that attenuation reflects the subsample rather than the added covariates. The age gradient was not an artifact of the linear form in which age enters: adding a quadratic age term left the interaction unchanged (*β* = −0.071, *p* = 0.012) and the quadratic term itself was non-significant (*p* = 0.726), as was its interaction with Instagram frequency (*p* = 0.912) when both were entered. Mode of administration did not alter the pattern: self-completion by link (39.5% of the analytical sample) was associated with higher reported distress (*β* = +0.050, *p* = 0.028), but, with the mode indicator added, every headline digital coefficient retained its direction and significance (Instagram *β* = +0.060, *p* = 0.022; WhatsApp *β* = −0.070, *p* = 0.009; social media time *β* = +0.090, *p* = 0.013; age × Instagram *β* = −0.068, *p* = 0.015; age × social media time *β* = +0.068, *p* = 0.024), and the mode × platform interactions were jointly non-significant (*F*(3, 1,455) = 0.43, *p* = 0.730; Supplementary Table S8).

Two exploratory ancillary analyses drew no support and are reported for transparency (Supplementary Table S9). ChatGPT/large-language-model (LLM) use, added to a reduced model (demographic controls, social media time, Instagram, WhatsApp, and loneliness; *N* = 1,308), showed no significant association (*β* = +0.037, *p* = 0.086) and has not yet crystallized as an independent risk indicator in the adult population. Additionally, neither a three-way gender × age × Instagram interaction (*β* = −0.026, *p* = 0.278) nor a female × Instagram interaction (*β* = +0.006, *p* = 0.826) reached significance, providing no evidence that the Instagram association is moderated by gender. To control the false discovery rate across the twenty-test hypothesis family in Table 3 and Supplementary Tables S8, S9, a Benjamini–Hochberg correction was applied. The core pattern held: loneliness, age, female gender, household income, social media time, Instagram, WhatsApp, the age × Instagram interaction, religiosity, lifetime unemployment experience, sleep duration, maternal closeness, and the regional NUTS-1 joint *F* test all remained significant (pFDR ranging from 0.003 to 0.027). The LLM coefficient (pFDR = 0.114) and the three-way interaction (pFDR = 0.327) stayed non-significant, while the age × social media time interaction remained significant after correction (*p* = 0.020, pFDR = 0.029).

Taken together, the study’s hypotheses were largely supported. Hypothesis 1 was supported after demographic adjustment (Block 2, *β* = +0.068, *p* = 0.029), while total internet time showed no association (*β* = −0.004; Section 3.2). The social media time *p*-value rose to 0.051 when platform frequencies were included (Block 3), and the coefficient settled at *β* = +0.088 in the full model, a value the wild-cluster bootstrap leaves short of 0.05 (*p* = 0.063). Hypothesis 2 was supported in its differentiated form: age acted as a confounder for social media time and Instagram and as a suppressor for WhatsApp, whose near-zero bivariate correlation (*r* = −0.028) gave way to a significant protective coefficient (*β* = −0.073; Table 3). The suppression half of this prediction was formed *post hoc* and is reported as exploratory. Hypothesis 3 was supported in direction, with Instagram positive, WhatsApp protective, and TikTok and YouTube not statistically significant, though all associations were small. Hypothesis 4 was supported, in the primary specification, by the age × Instagram interaction (*β* = −0.069, *p* = 0.014), and Hypothesis 5 by the two-factor measurement solution, whose configural invariance was supported only partially across gender and across the six age groups. The exploratory mediation question was not supported by the bootstrap test, as all three confidence intervals included zero. The rise in *R*^2^ from 0.127 to 0.359 (largely variance shared with the outcome battery; Section 3.2) identifies loneliness as a strong independent correlate with no detectable indirect role in the digital-use associations.

## 4 Discussion

### 4.1 Principal findings

The small-effect pattern from high-income settings holds in the Turkish adult population. Instagram carried *β* = +0.066, WhatsApp *β* = −0.073, and social media time *β* = +0.088, though the three do not carry equal evidential weight. Under the wild-cluster bootstrap, the two platform coefficients held (Instagram *p* = 0.036, WhatsApp *p* = 0.024), whereas the aggregate social media-time coefficient (*p* = 0.063) and its interaction with age (*p* = 0.067) did not. Additionally, the aggregate coefficient was the term most sensitive to the exposure-scale checks, moving from *p* = 0.005 under influence exclusion to 0.033 under winsorizing and 0.109 under the log transform (Section 3.3). They are of the same order as Orben and Przybylski’s estimates: their specification-curve analysis of three US and UK adolescent datasets (*n* = 355,358) found that digital-technology use explained at most 0.4% of the variance in wellbeing (45), against a combined Δ*R*^2^ of 0.011 for the two digital blocks here (0.0036 + 0.0078) (a different outcome, sample, and age range, so not a like-for-like comparison). Their time-use-diary work across Ireland, the UK, and the US returned null-to-trivial associations (3). Significance here reflects the large sample (*N* = 1,478); the magnitude stays below Cohen’s small-effect benchmark and warrants caution pending longitudinal replication. In practical terms, a one-standard-deviation difference in platform-use frequency (a seven-point scale) or in daily social media time (a 27-category ordinal rank, not a 27-point score) shifts predicted distress by under half a point on the 9-to-36 distress composite, which itself spans 27 points. This change is unlikely to be individually meaningful even where it is reliably detected at the population level, so the public health relevance rests on aggregation across a near-universal user base rather than on individual-level magnitude.

The small effect sizes admit three interpretations. The first treats social media use as a weak but statistically detectable population-level risk indicator. The second is measurement: given the self-report-to-logged correlation of about 0.38 reported by Parry and colleagues (8), and under the assumption of purely random measurement error, classical disattenuation implies a factor of roughly 1.6 times the observed coefficient. Because that meta-analysis could not establish the direction of the discrepancy (over- and under-reporting were equally common across comparisons, 46.9% each), this factor is best read as an illustrative bound rather than a correction, and it applies symmetrically to the protective WhatsApp coefficient and the null platforms, not only to the positive ones. The third interpretation is that average associations conceal subgroup differences, consistent with the developmental-susceptibility reading of the Differential Susceptibility to Media Effects model: the Instagram slope on psychological distress rises to *β* = +0.132 among young respondents. These readings coexist: the association is likely real, partly obscured by measurement, and unevenly distributed across age groups.

Compared with the three large-scale studies in Supplementary Table S10 (6, 7, 9), the present study relies on probability-based stratified cluster sampling rather than an online panel, an insurance-member panel, or national online quotas. However, it does not estimate prevalence, as it did not employ a clinical instrument or an impairment criterion. The divergent prevalence figures from the first two studies (0.75–1.2% versus 66.7%) illustrate how significantly case definitions influence such estimates, whereas behavioral indicators reflect population distribution. The platform-specific pattern aligns with evidence from other studies. In a cross-sectional analysis of the 2020 wave of the China Family Panel Studies, which included adults aged 60 and over, interpersonal messaging (WeChat) use was linked to lower depressive symptoms, whereas short-video use was associated with higher symptoms (unstandardized *B* = −0.96 and *B* = +1.24 for binary use indicators, not directly comparable in magnitude to the standardized coefficients reported here). This interpersonal versus content contrast parallels the WhatsApp protective pattern observed here, even though the short-video association there has no counterpart in the present sample’s null TikTok coefficient (46). Consistent with this, messenger use (which includes WhatsApp, Facebook Messenger, and Telegram) and video calling were significant negative predictors of depressive symptoms in a non-representative sample of 490 German smartphone users studied during the COVID-19 pandemic (47). The clearest contribution is the platform-specific associations (Instagram positive, WhatsApp negative), established in a national Turkish probability sample. The age gradient in the Instagram association and the aggregate social media time association are secondary to this finding, with the first because its interaction term only meets the conventional threshold in the loneliness-adjusted model and the second because the most demanding clustered test does not support it.

### 4.2 Platform-specific associations and their context

Not all screen time is equally associated with psychological distress. By design, algorithm-driven Instagram (*β* = +0.066, *p* = 0.011) and short-video TikTok stand on one side, while interpersonal messaging on WhatsApp (*β* = −0.073, *p* = 0.007) stands on the other, with YouTube’s mixed architecture in between; empirically, only the Instagram and WhatsApp coefficients reached significance. This pattern is consistent with reviews that locate social media effects in content and individual characteristics rather than in use intensity (17–19). Instagram operates on algorithmic content streams, visual perfection pressure, and upward comparison, while WhatsApp relies on end-to-end encryption and small-group or one-to-one messaging without an algorithmic feed. The contrasting outcomes align with accounts that attribute social media effects to platform architecture rather than a simple active–passive use mode, although the present measures index frequency and duration rather than mode of use, and a two-platform contrast cannot fully isolate architecture from other correlated differences. Sarman and Tuncay (12) found that Turkish adolescent WhatsApp and Telegram users scored lower on loneliness overall, even though those who primarily used these apps for chatting scored higher. That finding parallels the adult WhatsApp–distress association here in direction, although in the present sample, WhatsApp use was unrelated to the loneliness item (*r* = −0.003 in the analytical sample), so the convergence concerns platform direction rather than a loneliness mechanism. The two MENA problematic-use studies reviewed here report prevalence, with aggregate problematic-use–symptom associations in the systematic review (20) and socio-demographic and application-use correlates in the Saudi survey (21), but neither reports a platform-level contrast. The Arab Barometer-based chapter (10), which models platform type against wellbeing in a set of countries that does not include Türkiye, reports no clear evidence that any one platform type accounts for the wellbeing penalty associated with heavy use. However, among heavy users, those whose main purpose is private communication rate themselves markedly better off than their parents, a result only loosely convergent with the WhatsApp association here. None of the three is directly comparable to the present estimates.

Some 86.2% of individuals aged 16–74 in Türkiye reported using WhatsApp in the preceding 3 months (13), and the sample mean of 6.25 reflects that penetration. Three candidate explanations, none testable here, relate to the association: the persistence of kinship ties, with WhatsApp family groups plausibly serving as a digital extension of sıla-yi rahim, the religiously framed obligation to maintain kin contact that remains salient in Türkiye; the bridging of migration-driven distance, where WhatsApp facilitates family continuity; and minimal performative pressure, since a WhatsApp message exists outside the public approval cycle of an Instagram post. Because use is so close to the scale ceiling, the exposure range is narrow, and the WhatsApp coefficient relies largely on the contrast between the minority reporting less than daily use and the large majority reporting daily or more. This minority is not confined to respondents who never use the application, as the association remained negative even after they were excluded (Section 3.3). However, the compressed range leaves little scope to distinguish degrees of use above the daily threshold.

Instagram’s adverse direction is unlikely to reflect algorithmic delivery alone. Two cultural factors are salient in Türkiye: a tradition in which visual appearance and external presentation carry significant social evaluation, and a rapidly expanded consumption capacity over two decades intersecting with the visual display of class inequality on image-centered platforms. The influencer figure entered mainstream Turkish culture after 2010, likely making the ground for social comparison denser than in some Western contexts. These interpretations cannot be tested directly and are candidate explanations, not established mechanisms.

TikTok’s non-significant coefficient may seem surprising, but distribution is decisive: the TikTok mean is 2.45 on a seven-point scale, falling between “once a month or less” and “a few times a month,” and much of the adult population uses it seldom or not at all. Daily or more frequent use is concentrated among the youngest adults (35.1% of 18- to 24-year-olds versus 4.9% of those aged 65 and over in the analytical sample), whereas the sample’s mean age (39.53 years) is significantly higher than that group, resulting in a narrow informative range at the population level. The model shows no indication of an association obscured by that narrowness: the age × TikTok term is positive rather than negative (*β* = +0.030, *p* = 0.183), and the age-stratified estimates are weakest in the youngest group (*β* = +0.021; Supplementary Table S2). Whether an association would appear below age 18 lies outside this sample. The TikTok coefficient at Q90 is essentially zero and non-significant (*β* = −0.027, *p* = 0.603), providing no basis for inference about the shape of the relationship. YouTube’s null association, despite its widespread use, is consistent with its mixed architecture—search- and subscription-driven viewing rather than the image-centered algorithmic feed of Instagram—which suggests that the elevated distress signal is specific to the image-centered feed rather than to algorithmic delivery in general. In direct contrast, however, Instagram does not differ significantly from TikTok or YouTube (*p* = 0.184 and 0.180), so this interpretation relies on the pattern of individual coefficients rather than on a tested platform-by-platform difference.

### 4.3 Age moderation and additional correlates

For most digital-use measures, age behaves as a confounder rather than a suppressor. The bivariate correlation between social media time and psychological distress is *r* = 0.109 (Table 2); with age and the demographic set held constant, it attenuates to *β* = +0.068 (Block 2). Because age correlates negatively with digital use (*r* = −0.391 for social media time) and with distress burden (*r* = −0.209), it acts as a positive confounder inflating the bivariate coefficient, so adjustment attenuates rather than amplifies it. WhatsApp shows the opposite: an essentially zero bivariate correlation (*r* = −0.028) turns into a negative, significant multivariate coefficient (*β* = −0.073), a classic suppression in which the protective signal is masked at the bivariate level by age-confounded exposure. Because it emerges only after adjustment, the WhatsApp association is the most adjustment-contingent of the core findings, although it holds across the clustering and subscale robustness checks and attenuates in the unemployment-and-sleep model, which is fitted on a different rotation subsample sharing only 688 respondents with the analytical sample (Section 3.3).

The age × Instagram interaction reached the conventional threshold only once loneliness entered the model. Although the conditional Instagram slope declines with age and remains positive into the forties in both specifications (Supplementary Table S17), the age gradient in the Instagram association is linked to cohort differences in digital adoption. Younger cohorts came of age during the post-2010 spread of smartphones, while middle-aged and older cohorts encountered digital adoption in adulthood. As a result, younger respondents exhibit both heavier digital use and an elevated distress burden. The COVID-19 pandemic likely added a further layer, with adoption among older adults accelerating during 2020 and 2021. However, a single 2024 cross-section cannot observe a shift over time, and these data neither support nor rule it out. What they do show is a sharply higher penetration of algorithm-driven platforms among younger respondents. The loneliness-adjusted Johnson–Neyman threshold near 42–43 years, itself estimated only within a wide band (Section 4.4), falls between the descriptive turning point near 35, at which Chapter 8 of the 2026 World Happiness Report identifies a change of sign, and its Millennial–Gen X birth-year cutoff of 1980 and 1981, which we convert to ages 43–44 in 2024. Across this range, the cohort coefficient moves from moderately negative to near zero (22), while the loneliness-free threshold near 45 years lies just above that cutoff. In 2024, the 42-to-43-year point corresponds to the oldest Millennials (born 1981–1982), so the region below it encompasses Millennials and Gen Z, the two cohorts for which that chapter reports negative internet–wellbeing relationships.

Adding the loneliness item (welalone) raised *R*^2^ from 0.127 to 0.359, and loneliness emerged as the strongest single predictor (*β* = +0.498, *p* < 0.001). Because welalone is drawn from the same past-week emotional-state battery as the outcome, its correlation with the composite (*r* = 0.545) and part of this *R*^2^ gain reflect shared content and common-method variance rather than a fully distinct construct; the composite excludes welalone to avoid tautology, but residual overlap remains. The exploratory percentile bootstrap test, however, found no significant indirect pathway from any of the three digital predictors tested to psychological distress through loneliness; all three 95% confidence intervals included zero (Table 5). Loneliness is therefore best interpreted as a strong independent correlate with no detectable indirect pathway through the single-item measure; whether it lies upstream or downstream of digital use cannot be resolved in this cross-section.

**Table 5:** Hypothesis testing summary matrix.

Hypothesis	Prediction	Result	Evidence
H1	Social media use shows a small positive association with distress after demographic adjustment	Supported (Block-5 aggregate term clustering-sensitive)	Social media time *β* = +0.068 (Block 2) → +0.063, *p* = 0.051 once platform frequencies enter (Block 3) → +0.088 (Block 5); the Block-5 aggregate coefficient does not clear the wild-cluster bootstrap threshold (*p* = 0.063), and at *β* = +0.088 it falls below its own minimum detectable effect of 0.102 (Supplementary Table S13)
H2	Age operates as a confounder for most platforms and as a suppressor for WhatsApp	Supported (differentiated)	Social media time *r* = 0.109 → *β* = 0.068 (Block 2) → *β* = 0.088 (Block 5); Instagram *r* = 0.102 → *β* = 0.074 (Block 3) → *β* = 0.066 (Block 5); WhatsApp *r* = −0.028 → *β* = −0.081 (Block 3, suppression); the WhatsApp-suppression expectation was exploratory, formed *post hoc* after inspecting the bivariate structure
H3	Platform associations differ in direction	Supported in direction (Instagram and WhatsApp only; TikTok and YouTube null)	Instagram (+), WhatsApp (−); TikTok and YouTube non-significant
H4	Age moderates the Instagram association	Supported (specification-dependent)	Age × Instagram *β* = −0.069, *p* = 0.014 in Block 5 and *β* = −0.060, *p* = 0.059 in Block 4, so significant only after loneliness enters, and the two estimates are not distinguishable from one another (bootstrap difference −0.009, 95% CI [−0.041, +0.021]). The term clears the wild-cluster bootstrap (*p* = 0.044), the twenty-test FDR family (pFDR = 0.025), the seven-item outcome (*p* = 0.013) and a quadratic age control (*p* = 0.012), but not the five-item depressive subscale (*p* = 0.100), and at |*β*| = 0.069 it falls below its own minimum detectable effect of 0.078 (Supplementary Table S13)
H5	Two-factor structure fits in the Turkish adult sample (a measurement precondition)	Supported (configural invariance only partial across gender and age groups; metric invariance untested)	CFI = 0.950, RMSEA = 0.078
Exploratory question	Single-item loneliness carries a detectable indirect pathway from digital use to distress	Not supported by exploratory bootstrap test	Bootstrap indirect effects: Instagram *β* = +0.014 95% CI [−0.013, 0.042]; SM time *β* = +0.010 [−0.018, 0.039]; WhatsApp *β* = +0.003 [−0.026, 0.030]. Loneliness operates as independent predictor

Three secondary patterns among the digital main effects accompanied the addition of loneliness, each qualified by the unresolved role of loneliness set out in the Methods. Social media time strengthened from *β* = +0.078 to *β* = +0.088, with the adjustment moving it away from zero rather than toward it. The Instagram coefficient declined by 0.013 (from +0.079 in Block 4 to +0.066 in Block 5) while retaining significance (*p* = 0.011). Under a confounder interpretation, the Block-5 value is the cleaner estimate, whereas under a mediator interpretation, Block 4 provides the total association and Block 5 gives the direct one, making the Block-5 coefficient a lower bound under the mediator interpretation alone. A third interpretation sits alongside these two and is measurement rather than causal: the loneliness item is drawn from the same past-week battery as the outcome and loads on the same negative-affect factor in the confirmatory model (Section 3.1), so conditioning on it removes variance that the outcome measure itself carries. Under that reading, the Block-5 estimate is neither the cleaner specification nor a bound of any kind. The precision gain is affected in the same way: entering welalone lowers the residual standard deviation from 5.00 to 4.29 and shrinks the standard errors of the model’s coefficients by a median of 12%, including the age × Instagram term (11.6%). Thus, part of what carries that term across the conventional threshold is a model-wide reduction in residual variance obtained by including outcome-overlapping content among the predictors. The term suppression, which this manuscript reserves for the age-masked WhatsApp association reported above, does not describe this pattern. Because the cross-sectional design cannot adjudicate among the three interpretations (34), Block 4 and Block 5 are reported side by side throughout. Where a single verdict is required, as in the hypothesis summaries and Table 5, it is taken from Block 5 as the full specification, with the Block-4 result given alongside it. The bootstrap test, which cannot reject the mediator interpretation, does not settle the choice. The two age interactions moved in the opposite direction, with the age × Instagram term strengthening from *β* = −0.060 (*p* = 0.059) in Block 4 to *β* = −0.069 (*p* = 0.014) in Block 5, and the age × social media time term from *β* = +0.039 (*p* = 0.247) to *β* = +0.070 (*p* = 0.020). Therefore, the lower-bound reading available for the Instagram main effect under the mediator account does not transfer to them. Under a mediator interpretation, the Block-4 terms carry the total moderation and the Block-5 terms are not conservative estimates, whereas under a confounder interpretation, Block 5 remains the cleaner specification. The two interaction estimates are also indistinguishable from one another: a percentile bootstrap of their difference (2,000 resamples) returns −0.009 with a 95% confidence interval of [−0.041, +0.021], indicating that the blocks differ in which side of the conventional threshold the term falls on rather than in the size of the moderation they estimate. The WhatsApp coefficient held its protective direction almost unchanged (*β* = −0.073, *p* = 0.007), suggesting that the WhatsApp association is not accounted for by loneliness. In the displacement–augmentation terms of the Introduction, the protective WhatsApp direction is consistent with augmentation, though the absence of a detectable loneliness pathway leaves the mechanism untested. The never-married association, by contrast, was absorbed by the loneliness adjustment (from *β* = +0.058, *p* = 0.071 in Block 4 to *β* = +0.003, *p* = 0.920 in Block 5) and is therefore not independent of loneliness. However, as noted in the Results, whether loneliness mediates or confounds that link cannot be separated in this cross-section.

The exploratory test of whether young women experience the Instagram association more strongly was unsupported (three-way interaction *β* = −0.026, *p* = 0.278; female × Instagram *β* = +0.006, *p* = 0.826): no gender moderation was detectable, so the boundary this design can locate is the age gradient rather than a gender contrast, and the absence of a detectable gender difference does not prove that none exists. Women’s higher symptom burden overall (*β* = +0.113) is confirmed but represents a general gender gap across platforms rather than an Instagram-specific phenomenon. The exploratory test of whether LLM use functions as a digital risk indicator was likewise unsupported (*β* = +0.037, *p* = 0.086; pFDR = 0.114). LLM adoption in 2024 was at an early diffusion stage, with 72.4% of respondents reporting no use at all and a further 6.4% using it less than monthly (78.8% combined), compressing variance and possibly attenuating any association; the borderline positive direction should not be over-interpreted.

### 4.4 Theoretical implications and policy questions for future research

Most evidence on digital use and mental health comes from adolescent and young-adult samples (3, 11, 12); testing the Differential Susceptibility to Media Effects model (16) in a nationally representative Turkish adult sample suggests an approximate age boundary near the early forties. Only developmental (age) susceptibility is probed here, and within a single cross-section, developmental susceptibility and a secular cohort or adoption gradient are observationally equivalent. The 18-to-43 window from the loneliness-adjusted model encompasses, in 2024, all Millennials (1981–1996) and the adult portion of Gen Z (born 1997–2006), and the threshold offers a testable prediction for other upper-middle-income settings with comparable digital-adoption gradients. However, it marks where the conditional Instagram slope becomes statistically indistinguishable from zero at this sample size rather than where its point estimate reaches zero (near 54 years in the loneliness-adjusted model). That location is loosely determined: carrying the age × Instagram coefficient across its own 95% confidence interval (Table 3) shifts the zero-crossing from about 48 years to beyond the observed age range, so the boundary should be understood as an approximate region in the middle of the adult age range rather than as a specific point. Within Türkiye, religiosity entered only as a population-level covariate (*β* = −0.085), with the three religiosity × platform interactions jointly non-significant (*F*(3, 2,159) = 0.98, *p* = 0.399; Supplementary Table S8), indicating no evidence in these data that the platform pattern varies with individual religiosity. Kardefelt–Winther’s (5) Compensatory Internet Use framework, developed at the individual-difference level, treats loneliness as an antecedent of use rather than a downstream consequence; this cross-section cannot estimate that direction, and a formal test would require psychosocial needs indicators that the 2024 wave does not provide. Loneliness added substantially to explained variance as a strong population-scale correlate, although the bootstrap test detected no pathway running from use through loneliness to distress. That test bears on the displacement direction; the compensatory direction that the framework posits was not testable here. WhatsApp’s protective-direction association suggests that interpersonal communication platforms complement, rather than substitute for, the social support function, though the present data show no direct loneliness pathway.

Two questions for future research arise from these patterns, neither of which the present design can support. First, whether digital wellbeing work attentive to platform type performs differently from work that treats screen time as a single quantity is open to longitudinal or experimental testing, with media-literacy work oriented to algorithm-driven image platforms (Instagram) among younger adults as the most direct test case. The small magnitudes argue against expecting large individual-level differences even under confirmation. Second, the protective-direction association of interpersonal messaging use is best treated as a longitudinally testable hypothesis rather than an actionable prescription: the cross-sectional design cannot establish it as a mechanism or confirm its direction, and within age bands, it reached significance only among 30- to 49-year-olds (Section 3.2).

The five core hypotheses were supported with qualifications: the Block-5 aggregate social media time coefficient behind H1 does not meet the wild-cluster bootstrap threshold (*p* = 0.063); the suppression component of H2 was formed *post hoc* rather than in advance and is therefore exploratory; the platform associations behind H3 were small and limited to Instagram (positive) and WhatsApp (negative), with TikTok and YouTube showing null results; the age × Instagram moderation behind H4 reached significance only in the loneliness-adjusted specification; and the two-factor solution behind H5 met the lenient rather than the strict CFI and RMSEA thresholds, with its configural invariance holding only partially across gender and the six age groups, while metric invariance was untestable at these subgroup sizes. The conceptual framework matched the structure of the data with two qualifications: the categorical prediction behind H3 held only for Instagram among the algorithm-driven platforms, and the exploratory mediation chain through loneliness was not statistically detectable with the single-item measure available (Table 5). In the Turkish adult population, the association between digital use and psychological distress is small, varies in sign by platform, and differs in strength with age; no single mechanism accounts for this pattern. By anchoring these patterns in a national probability sample from a Muslim-majority, non-Arab, upper-middle-income country, the study extends population-level evidence on digital use and psychological distress beyond the high-income Western settings that dominate the literature.

### 4.5 Limitations and generalizability

The design is cross-sectional, so causal inference is limited; the Instagram association can be interpreted in reverse, with more distressed individuals engaging more intensely with Instagram and withdrawing from interpersonal messaging. Digital-use measures rely on self-report, which correlates only about 0.38 with logged use (8); under a random-error assumption, this attenuates effect sizes, though the meta-analysis could not determine the direction of the discrepancy. Each headline coefficient also sits at the limit of what this design could detect, being significant at 0.05 but not at 0.005, as the minimum detectable effects reported in the Methods and in Supplementary Table S13 indicate. At that level of precision, an estimate that reaches conventional significance is on average larger in magnitude than the association it estimates. This upward pressure on magnitude works against the measurement attenuation just noted rather than adding to it. Because neither can be shown here to leave a larger mark, the coefficients are best interpreted without correction in either direction. Neither bias affects the direction of the associations, which the clustered and multiverse checks support. The sample is restricted to adults (18+) and cannot be generalized to those under 18. No clinical instrument (e.g., SAS-SV, BSMAS) was administered, since TGSS is a general-purpose social survey not designed as a problematic-use module; behavioral indicators cannot estimate clinical-level prevalence or capture the functional-impairment criterion. Compared to a purpose-designed study, this limits measurement depth, a cost offset by the gain in probability-based national coverage. A measurement constraint also affects the age moderation. Configural fit was only partially adequate across the six age groups, and metric invariance could not be tested at these subgroup sizes (Section 3.1), so age-varying measurement of the composite remains an alternative interpretation of the age × Instagram interaction that these data cannot exclude. On the outcome side, the age × Instagram moderation is sensitive to how the distress index is composed. Re-fitting the model on the five-item CES-D-mapped depressive subscale preserved the platform associations and the age × social media time interaction, but returned the age × Instagram interaction at *β* = −0.049, *p* = 0.100 (Supplementary Table S14). In contrast, restricting the outcome to the seven negatively worded items retained it (*β* = −0.072, *p* = 0.013), a construction in which the age × social media time interaction in turn fell short (*β* = +0.054, *p* = 0.077; Section 3.3). The generality of the early-forties boundary is therefore limited by how the outcome index is composed, and because the difference between these fits was not itself tested and is compatible with the lower reliability and shorter length of the five-item subscale (*α* = 0.778 versus 0.875 for the nine-item index), it should not be interpreted as locating the moderation in one symptom domain. Because the 2024 collection followed pandemic restrictions that reshaped digital use, average use levels may be inflated relative to a pre-pandemic baseline. The dataset captures a Muslim-majority, upper-middle-income, non-Arab country, but generalization to other Muslim-majority settings (particularly the Arab Gulf and South Asia) or lower-middle-income contexts would require independent replication. The loneliness pathway relied on the single-item welalone indicator within the CES-D-informed battery; a multi-item instrument, such as Hughes and colleagues’ three-item scale (23), would offer richer coverage and a better-powered mediation test, so the negative bootstrap result should be interpreted as limited by the single-item measure. The public-use release also provides neither design weights nor address-block cluster identifiers, so the standard errors of the main HC3-based specification do not adjust for design-based clustering. Recruitment in public spaces can under-represent adults who rarely leave home, and the realized sample has 7.8% aged 65 and over compared to 14.3% in the population register, which the age and gender controls and the post-stratification raking sensitivity in Supplementary Table S7 address on the representation side. The implied design effect of 1.21 is a weighting (Kish) design effect that captures only variance inflation from unequal post-stratification weights; it does not account for the clustering design effect from the 134 address-block clusters. Clustering the standard errors on the 26 NUTS-2 subregions, the finest geographic level in the public file, increased those of the five digital coefficients by 3–7% and left the headline coefficients significant (Supplementary Table S13). In contrast, the more demanding wild-cluster bootstrap left the platform contrasts and the age × Instagram interaction significant, while the aggregate social media time terms fell short (Section 3.3; Supplementary Table S15). Because intra-cluster correlation at the address-block level can exceed that at the subregional level, residual underestimation of the standard errors cannot be ruled out. Finally, the survey combined interviewer-administered and self-completed modes; self-completion was associated with somewhat higher reported distress, but the platform coefficients changed little and retained their direction and significance when mode was controlled for (Section 3.3).

## 5 Conclusion

In this national probability sample of Turkish adults, the study’s strongest contribution is that screen time is not uniform in its association with psychological distress: Instagram and WhatsApp, the sample’s principal algorithm-driven image platform and interpersonal messaging application, are associated with psychological distress in opposite directions. This contrast is consistent with, though not a full test of, the algorithm-driven versus interpersonal distinction. Instagram (*β* = +0.066, *p* = 0.011) functioned as a small positive risk indicator, while WhatsApp (*β* = −0.073, *p* = 0.007) showed a small inverse (protective) association with psychological distress. Overall social media time (*β* = +0.088, *p* = 0.016) was also positively associated, though less firmly held, while TikTok and YouTube yielded no detectable independent association. Platform composition, rather than overall exposure, carried the signal that survived the most demanding clustering and exposure-scale checks, indicating that treating social media use as a single homogeneous category is inadequate at the population level.

The age-by-Instagram interaction (*β* = −0.069, *p* = 0.014) and the Johnson–Neyman analysis located a threshold of approximately 42–43 years, beyond which the Instagram association was indistinguishable from zero. This finding concentrates the Instagram association among younger adults and provides the Differential Susceptibility to Media Effects framework (16) with an approximate population-level age boundary in the early forties, which a single cross-section cannot separate from a cohort or adoption gradient. The interaction reached the conventional threshold only once loneliness entered the model, and the boundary is loosely determined: across the interaction coefficient’s own confidence interval, the age at which the slope reaches zero varies from about 48 years to beyond the observed range (Section 4.4). Therefore, it is a secondary, specification-dependent finding that marks a region rather than a specific point in time. The two-factor negative- and positive-affect structure of the 10-item CES-D-informed battery fit the Turkish adult data adequately (CFI = 0.950, RMSEA = 0.078). Adding loneliness raised the explained variance from 0.127 to 0.359, an increment that largely reflects variance shared with the outcome battery, while the digital coefficients remained significant. Loneliness acted as a strong independent correlate, and the single-item measure detected no indirect pathway from use through loneliness to distress, a result that bears on the displacement direction. The compensatory direction posited by the Compensatory Internet Use view, in which loneliness precedes rather than follows use, was not testable here (5).

Drawn from the openly accessible TGSS 2024 wave, these findings provide platform-specific population evidence for a Muslim-majority, non-Arab, upper-middle-income population that falls outside the Arab Barometer (10) and is represented in multi-country online panels only by a single national quota of internet users without platform-level detail (9). Given the small individual-level associations, whether population-level digital wellbeing work is better at distinguishing platform types than at reducing screen time uniformly is a question these findings raise for longitudinal and experimental research rather than a conclusion they establish. The protective-direction association of interpersonal messaging use is in the same position: a hypothesis for longitudinal testing rather than an immediate prescription.

## Data Availability

The datasets presented in this study can be found in online repositories. The names of the repository/repositories and accession number(s) can be found at: The Turkish General Social Survey (TGSS) 2024 dataset is publicly available in the Zenodo repository at: https://doi.org/10.5281/zenodo.18721350 (accession/DOI: 10.5281/zenodo.18721350).
