# Supplementary material for: Platform-specific digital media use and psychological distress among Turkish adults: evidence from a nationally representative, cross-sectional survey
Source: Front Public Health. 2026 Sep 21;14:1941229. doi: 10.3389/fpubh.2026.1941229 (PMC13635173; doi:10.3389/fpubh.2026.1941229)
Supplement: Supplementary file 1 [file Supplementary_file_1.DOCX]

**Supplementary Material**

**Platform-specific digital media use and psychological distress among Turkish adults: evidence from a nationally representative, cross-sectional survey**

**Table S1. Confirmatory factor analysis fit indices**

| **Model** | ***χ²*** | ***df*** | **CFI** | **TLI** | **RMSEA** | **SRMR** |
| --- | --- | --- | --- | --- | --- | --- |
| Single-factor (Model A) | 1,051.25 | 35 | .894 | .864 | .111 | .055 |
| Two-factor (Model B) | 516.19 | 34 | .950 | .934 | .078 | .035 |

*Note. Both models were estimated with maximum likelihood in the Python semopy package on the 2,358 respondents with complete data on the ten-item battery. Model A loads all ten items on a single factor; Model B loads the eight negatively worded items on a negative-affect factor and the two positively worded items, entered reverse-coded, on a positive-affect factor. The two-factor model fits significantly better by the chi-square difference test (Δχ²(1) = 535.06, p < .001).*

**Table S2. Age-stratified regression results (standardized coefficients, nine-item mean composite dependent variable, DV)**

| **Variable** | **18-29 years (*N* = 451)** | **30-49 years (*N* = 649)** | **50+ years (*N* = 378)** |
| --- | --- | --- | --- |
| Social media time | +.008 | +.057 | +.173* |
| Total internet time | +.080 | -.029 | -.098 |
| TikTok frequency | +.021 | +.048 | +.060 |
| Instagram frequency | +.091† | +.112** | -.042 |
| YouTube frequency | -.001 | +.009 | -.012 |
| WhatsApp frequency | -.003 | -.098** | -.131† |
| *R*² | .060 | .125 | .121 |

*Note. †p < .10. *p < .05. **p < .01. Demographic controls (age, gender, education, household income, marital indicator, household size) included in each model; standardized coefficients computed within each subsample using the nine-item distress composite (mean × 9) as the dependent variable. Loneliness and the age × platform interaction terms were not included. The p-values are uncorrected and lie outside the twenty-test FDR family (Table S16).*

**Table S3. Quantile regression coefficients (standardized, nine-item DV)**

| **Variable** | **Q25** | **Q50** | **Q75** | **Q90** |
| --- | --- | --- | --- | --- |
| Social media time | +.055 | +.045 | +.054 | +.128* |
| Instagram | +.114** | +.068* | +.065 | +.114* |
| TikTok | +.043 | +.030 | +.029 | -.027 |
| YouTube | -.008 | +.043 | +.009 | +.007 |
| WhatsApp | -.070 | -.123*** | -.083* | -.076 |
| Age | -.132** | -.172*** | -.130* | -.144* |
| Female | +.211*** | +.121*** | +.142*** | +.093 |

*Note. *p < .05. **p < .01. ***p < .001. Female coefficients reported as standardized β for cross-quantile comparability. Quantile models included the demographic controls and digital-use measures but not the age × platform interactions or the loneliness covariate. The p-values are uncorrected and lie outside the twenty-test FDR family (Table S16).*

**Table S4. Latent profile analysis (k = 3 selected for interpretability; LPA estimated on the broader N = 2,311 sample with valid platform indicators; seven standardized indicators: daily social-media time and TikTok, Instagram, YouTube, WhatsApp, Twitter, and Facebook frequency, listwise with the outcome, age, and gender)**

| **Profile** | ***N*** | **%** | **Description** |
| --- | --- | --- | --- |
| Profile 1: Heavy multi-platform | 798 | 35 | High use across all platforms, active Twitter use |
| Profile 2: Moderate, non-Twitter | 1,091 | 47 | WhatsApp highest, low Twitter use |
| Profile 3: Low use | 422 | 18 | Low use across all platforms |

*Note. One-way ANOVA across profiles for the nine-item psychological-distress composite reached F = 1.07, p = .342, which was not significant. BIC model-selection diagnostics for k = 2 to 6 are reported in Supplementary Table S5.*

**Table S5. LPA model-selection diagnostics (k = 2 to 6, indicators standardized prior to estimation)**

| **k** | **BIC** | **AIC** | **Entropy** | **Smallest class %** |
| --- | --- | --- | --- | --- |
| 2 | 23,479.7 | 23,313.1 | .992 | 38.6 |
| **3 (selected)** | **9,400.6** | **9,147.8** | **.996** | **18.3** |
| 4 | -4,868.5 | -5,207.5 | .999 | 5.7 |
| 5 | -8,766.3 | -9,191.5 | .995 | 7.8 |
| 6 | -15,317.2 | -15,828.5 | .947 | 5.5 |

*Note. Although BIC and AIC continued to decrease for k ≥ 4, the smallest class fell below 8% from k = 4 onward, which produced classes too small for substantive interpretation. The steep decline in BIC and AIC for k ≥ 4 reflects additional near-degenerate components with almost zero within-class variance on the discrete, strongly zero-inflated indicators (in the k = 3 solution the non-Twitter and low-use profiles both have a Twitter mean of exactly 1.00) rather than substantively distinct additional profiles. Gaussian mixture model (GMM) estimation used a fixed random seed. The k = 3 solution was therefore selected on interpretability grounds despite a higher BIC, following the convention in applied LPA work to balance statistical and substantive criteria.*

**Table S6. Missing-data sensitivity analysis: coefficient comparison (nine-item DV)**

| **Variable** | **Listwise (*β*, *N* = 1,478)** | **Mean imputation (*β*, *N* = 2,564)** | **Absolute difference** |
| --- | --- | --- | --- |
| Social media time | +.063 | +.053 | .010 |
| Instagram | +.074 | +.049 | .025 |
| WhatsApp | -.081 | -.051 | .031 |
| Age | -.142 | -.157 | .015 |
| Female | +.144 | +.155 | .011 |

*Note. Coefficients retained direction under both methods; platform magnitudes attenuated under mean imputation (method-dependent magnitude). Both columns report Block-3 coefficients (demographics, total exposure, and platform frequencies; no interaction terms or the loneliness covariate).*

**Table S7. Sensitivity matrix: Listwise (main) vs multiple imputation by chained equations (MICE) pooled vs ADNKS-raked weighted Block 5 coefficients**

| **Variable** | **Listwise β (N = 1,478)** | **MICE pooled β (N = 2,564, m = 20)** | **ADNKS-raked β (effective N ≈ 1,223)** |
| --- | --- | --- | --- |
| Social media time | +.088* | +.056* | +.111* |
| Instagram | +.066* | +.040* | +.068* |
| WhatsApp | -.073** | -.052* | -.075* |
| Age × Instagram | -.069* | -.048* | -.098* |
| Age × Social media time | +.070* | +.033 | +.093* |
| Loneliness | +.498*** | +.515*** | +.480*** |
| Female | +.113*** | +.114*** | +.108*** |
| Age | -.110*** | -.139*** | -.122** |
| Block 5 R² | .359 | .374 | .344 |

*Note. †p < .10. *p < .05. **p < .01. ***p < .001. The MICE column reports pooled estimates under Rubin's (30) rules from m = 20 imputed datasets generated via scikit-learn IterativeImputer; the raked column reports weighted least squares (WLS) estimates after iterative proportional fitting to 2024 ADNKS marginals (age × gender, NUTS-1), using the exact 2024 ADNKS adult-population (aged 18 and over) shares computed from the TÜİK province-by-single-age-and-sex table (targets, age × gender: 18–29 men .1233, women .1183; 30–49 men .1976, women .1944; 50–64 men .1112, women .1124; 65 and over men .0637, women .0790; NUTS-1: TR1 .1877, TR2 .0479, TR3 .1350, TR4 .1035, TR5 .1009, TR6 .1258, TR7 .0491, TR8 .0586, TR9 .0336, TRA .0236, TRB .0419, TRC .0923); the raking converged within ten iterations, weights ranged from 0.52 to 4.07, and the Kish design effect was 1.21 (effective N ≈ 1,223). The age × social-media-time coefficient in the raked column has p = .0500, below .05 only at the fourth decimal, and is starred on that basis. Multiple imputation used IterativeImputer with BayesianRidge, sample_posterior = True, and a distinct random seed per imputation; the raked estimates are weighted least squares with HC3 standard errors under the raking weights. Numbered citations in this document follow the reference list of the main manuscript.*

**Table S8. Additional models summary matrix**

| **Model** | ***N*** | ***R*²** | **Added variable (*β* / *p*)** | **Comment** |
| --- | --- | --- | --- | --- |
| Religiosity | 2,178 | .384 | religious β = -.085, *p* < .001; godimp and pray insignificant | Digital coefficients preserved (digsmhr β = +.054, p = .015; Instagram β = +.042, p = .041; WhatsApp β = -.046, p = .024) |
| Region (NUTS 1) | 2,305 | .388 | NUTS F = 2.10, p = .017; Urban F = 2.00, p = .135 | East-West differentiation stronger than urban-rural; digital coefficients preserved (Instagram β = +.039, p = .045; WhatsApp β = -.052, p = .008) |
| Lifetime unemployment experience and sleep duration | 1,463 | .404 | unempev β = +.068, p = .001; sleephour β = -.080, *p* < .001 | Instagram and WhatsApp non-significant both before (p = .21 and .35) and after (p = .28 and .25) the added variables enter; the attenuation reflects the different rotation subsample (688 respondents shared with the analytical sample; the Instagram and WhatsApp point estimates are much smaller there, β = +.030 and -.022 before the added variables enter versus +.066 and -.073 in the analytical sample), not the added variables |
| Maternal emotional closeness | 733 | .412 | maclose β = -.131, *p* < .001; mavideo, maphone, matext insignificant | Quality of closeness matters more than communication frequency |
| Mode of administration (self-completion by web link vs face-to-face interview) | 1,478 | .361 | link β = +.050, p = .028 | Digital coefficients preserved (Instagram β = +.060, p = .022; WhatsApp β = -.070, p = .009; social-media time β = +.090, p = .013; age × Instagram β = -.068, p = .015; age × social-media time β = +.068, p = .024) |

*Note. The four contextual models differ in analytical sample and predictor set; none of them includes total internet time or the five age × platform interactions. The religiosity model (N = 2,178) retains social-media time and all four platform frequencies; the region (N = 2,305) and unemployment-and-sleep (N = 1,463) models retain social-media time with Instagram and WhatsApp only; the unemployment-and-sleep and maternal-closeness models omit household size (five demographic controls); all five models include the loneliness covariate; and the maternal-closeness model (N = 733) additionally swaps the media-use predictors for family-communication measures (mother-directed video, phone, and text contact). Sample sizes vary with the missingness of each added variable and with the questionnaire-rotation form on which it was fielded; the unemployment-and-sleep subsample shares 688 respondents with the analytical sample. The Comment column summarizes the consequence of each addition for the digital coefficients or the pattern among the added variables. A supplementary moderation check added religious × (social-media time, Instagram, WhatsApp) interaction terms to the religiosity model (N = 2,178); the three terms were jointly non-significant, F(3, 2159) = 0.98, p = .40. The mode-of-administration row differs from the other rows in using the full headline Block-5 specification (N = 1,478) with a self-completion indicator added (1 = questionnaire completed through a web link, 0 = face-to-face interview); in a second step its interactions with social-media time, Instagram, and WhatsApp were added and were jointly non-significant, F(3, 1455) = 0.43, p = .73. The religiosity-moderation and mode checks are reported outside the twenty-test FDR family.*

**Table S9. Two additional hypotheses without support**

| **Hypothesis** | ***N*** | ***R*²** | **Tested coefficient (*β* / *p*)** | **Result** |
| --- | --- | --- | --- | --- |
| LLM use is an independent risk indicator | 1,308 | .347 | digillm β = +.037, p = .086 | Not significant (p = .086; pFDR = .114) |
| Gender × Age × Instagram interaction | 2,305 | .381 | fem × age × insta *β* = -.026, p = .278; fem × insta *β* = +.006, p = .826 | Three-way and two-way interactions insignificant |

Note. LLM model: the six demographic controls (including household size), social-media time, Instagram, WhatsApp, LLM-use frequency, and loneliness (N = 1,308; total internet time and interaction terms not included). Three-way model: the same predictor set without LLM-use frequency, plus age × Instagram, female × Instagram, female × age, and female × age × Instagram (N = 2,305).

**Table S10. The present study alongside large-scale studies of problematic smartphone and social media use**

| **Dimension** | **Türkiye (this study)** | **USA (6)** | **France (7)** | **Ithra 30 countries (9)** |
| --- | --- | --- | --- | --- |
| Sample *N* | 1,478 (analytical) | 1,989 | 21,244 | 15,000 (500 per country) |
| Design | Probability stratified cluster | Online panel (Prolific) | Insurance member panel | Online panel |
| Clinical instrument | None (behavioral indicators) | SABAS | SCT | Adapted BSMAS/SMD items |
| Problematic smartphone/social-media use (PSU/PSMU) prevalence | Not estimated | 0.75-1.2% | 66.7% (8,025 of 12,034 screened) | 6.82% average |
| Social media time association | *β* = +.088 | Not reported | Not reported | 2+ h/day doubles PSMU odds |
| Instagram positive association | *β* = +.066 | Not reported | Not reported | Not reported |
| WhatsApp protective association | *β* = -.073 | Not reported | Not reported | Not reported |
| Age threshold (Instagram) | 42-43 years (loneliness-adjusted) | Not reported | Not reported | Not reported |
| Female elevated distress | *β* = +.113 | Not reported | Not reported | Not reported |

Note. Türkiye is itself one of the 30 nations in the Ithra sample (n = 385 Turkish social-media users among the national sample of 500; Turkish PSMU prevalence 7.3%, against 6.82% across the 30 nations); that study reports prevalence and socio-demographic correlates of PSMU, not platform-specific distress associations. Numbered citations in this document follow the reference list of the main manuscript.

**Table S11. Item-by-item correspondence between the TGSS past-week emotional-state battery and the ten-item CES-D (Andresen)**

| **TGSS item (wel)** | **English gloss** | **Correspondence to CES-D-10** |
| --- | --- | --- |
| weldepp | Felt depressed | Exact (CES-D-10: felt depressed) |
| welsleep | Restless / could not sleep well | Exact (restless sleep) |
| welhard | Everything felt an effort | Exact (everything an effort) |
| welhappy (reverse) | Felt happy | Exact (was happy; reverse-scored) |
| welalone | Felt lonely | Exact (felt lonely); used as loneliness predictor, excluded from composite |
| welstop | Could not get going with tasks | Approximate (could not get going) |
| welsad | Felt sad | CES-D-20 item; not part of CES-D-10 |
| welenjoy (reverse) | Enjoyed life | CES-D-20 item; not part of CES-D-10 |
| welworry | Could not stop or control worrying | Aligned with generalized-anxiety content (cf. GAD-7); not a CES-D item |
| welover | Could not overcome problems | Aligned with perceived stress (cf. PSS); not a CES-D item |

*Note. Four CES-D-10 items have no counterpart in this battery: bothered by things, trouble concentrating, hopeful about the future (reverse), and felt fearful. The composite is therefore CES-D-informed rather than a validated CES-D administration.*

**Table S12. Comparison of the analytical sample (N = 1,478) with the full survey sample (N = 2,615)**

| **Characteristic** | **Full sample (N = 2,615)** | **Analytical sample (N = 1,478)** |
| --- | --- | --- |
| Age, M (SD) | 40.3 (15.3) | 39.5 (14.9) |
| Female, % | 49.1 | 48.4 |
| Education (1–8 ordinal), M | 3.85 | 3.94 |
| Household income (1–24 ordinal), M | 10.42 | 10.53 |

*Note. The analytical sample closely tracks the full sample, with a mild skew toward slightly younger, more educated, and higher-income respondents consistent with platform-use coverage. Listwise-complete analytical sample on all model variables.*

**Table S13. Cluster-robust standard errors at the NUTS-2 level versus the HC3 main specification (Block 5, nine-item psychological-distress index, N = 1,478)**

| **Predictor** | **β** | **HC3 p** | **Cluster-robust p (NUTS-2)** | **SE ratio** | **Minimum detectable \|β\|** |
| --- | --- | --- | --- | --- | --- |
| Social-media time | +.088 | .016 | .024 | 1.07 | .102 |
| Instagram | +.066 | .011 | .015 | 1.05 | .073 |
| WhatsApp | -.073 | .007 | .010 | 1.04 | .076 |
| Age × Instagram | -.069 | .014 | .018 | 1.04 | .078 |
| Age × social-media time | +.070 | .020 | .024 | 1.03 | .085 |
| Loneliness | +.498 | <.001 | <.001 | 0.87 | — |

*Note. Cluster-robust standard errors computed on the 26 NUTS-2 subregions (mean ≈ 57 respondents per cluster), the finest geographic level in the public-use file. The final column reports the smallest standardized coefficient this design could detect with 80% power at a two-tailed .05 level, computed from each coefficient's own standard error, which the Block-5 confidence intervals in Table 3 imply; every headline coefficient falls below its own threshold. Standard errors of the five digital coefficients inflate by 3–7% (the loneliness standard error is slightly smaller under clustering, ratio 0.87) and every headline coefficient remains significant. The finer address-block cluster identifier is not released, so residual within-cluster correlation cannot be fully excluded.*

**Table S14. Sensitivity analysis on a five-item CES-D-mapped depressive subscale versus the nine-item psychological-distress index (Block 5)**

| **Predictor** | **Nine-item index β (p)** | **Five-item CES-D subscale β (p)** |
| --- | --- | --- |
| Social-media time | +.088 (.016) | +.090 (.013) |
| Instagram | +.066 (.011) | +.057 (.039) |
| WhatsApp | -.073 (.007) | -.057 (.043) |
| Age × Instagram | -.069 (.014) | -.049 (.100) |
| Age × social-media time | +.070 (.020) | +.067 (.034) |
| Loneliness | +.498 (<.001) | +.441 (<.001) |

*Note. The five-item CES-D-mapped depressive subscale comprises depressed mood, everything an effort, restless sleep, could-not-get-going, and (reverse-scored) happy (Cronbach's α = .778; r = .949 with the nine-item index, both computed on the full measurement sample; α = .783 and r = .951 on the analytic subsample). The composite is the mean of valid items × 5, requiring at least four of the five items (N = 1,472; this sample is not nested within the nine-item analytical sample: 1,469 respondents are shared, three respondents have a valid five-item but not a valid nine-item composite, and nine analytic respondents lack the five-item composite). The platform associations hold, whereas the age × Instagram moderation is weaker and non-significant on the subscale (β = -.049, p = .100); that difference is not itself tested and is compatible with the lower reliability (α = .778 versus .875) and shorter length of the subscale, so it should not be read as locating the moderation in one symptom domain.*

**Table S15. Wild-cluster bootstrap p-values for the five key digital coefficients versus HC3 and NUTS-2 cluster-robust p-values (Block 5, nine-item psychological-distress index, N = 1,478)**

| **Predictor** | **β** | **HC3 p** | **Cluster-robust p (NUTS-2)** | Wild-cluster p |
| --- | --- | --- | --- | --- |
| Social-media time | +.088 | .016 | .024 | .063 |
| Instagram | +.066 | .011 | .015 | .036 |
| WhatsApp | -.073 | .007 | .010 | .024 |
| Age × Instagram | -.069 | .014 | .018 | .044 |
| Age × social-media time | +.070 | .020 | .024 | .067 |

*Note. Restricted wild-cluster bootstrap-t with Rademacher weights, 9,999 replications, clustering on the 26 NUTS-2 subregions, with the null imposed on each coefficient in turn (seed fixed for reproducibility); this is the standard small-cluster remedy when the number of clusters is limited. The two platform contrasts (Instagram, WhatsApp) stay significant and the age × Instagram interaction remains significant (p = .044), whereas the aggregate social-media-time effect (p = .063) and the age × social-media-time interaction (p = .067) no longer clear .05, identifying the social-media-time main effect and its interaction with age as the most clustering-sensitive terms. HC3 and NUTS-2 cluster-robust p-values reproduce Table S13 for comparison.*

**Table S16. The twenty-test Benjamini–Hochberg FDR family (raw and corrected p-values, m = 20)**

| Test | Raw p | pFDR |
| --- | --- | --- |
| Loneliness (welalone, Block 5) | < .001 | = .003 |
| Female (Block 5) | < .001 | = .003 |
| Maternal closeness (S8) | < .001 | = .003 |
| Household income (Block 5) | < .001 | = .003 |
| Religiosity (S8) | < .001 | = .003 |
| Sleep duration (S8) | < .001 | = .003 |
| Age (Block 5) | < .001 | = .003 |
| Lifetime unemployment experience (S8) | = .001 | = .003 |
| WhatsApp (Block 5) | = .007 | = .016 |
| Instagram (Block 5) | = .011 | = .022 |
| Age × Instagram (Block 5) | = .014 | = .025 |
| Social-media time (Block 5) | = .016 | = .026 |
| NUTS-1 regional joint F (S8) | = .017 | = .027 |
| Age × social-media time (Block 5) | = .020 | = .029 |
| LLM use (S9) | = .086 | = .114 |
| Urbanization joint F (S8) | = .135 | = .169 |
| Gender × age × Instagram (S9) | = .278 | = .327 |
| TikTok (Block 5) | = .457 | = .508 |
| YouTube (Block 5) | = .623 | = .656 |
| Never-married (Block 5) | = .920 | = .920 |

Note. Raw p-values below .001 entered the correction at the conservative cap of .001, per the rule stated in the Methods; corrected values were computed from unrounded raw p-values. Family selection rule: one test per construct, namely the five platform and social-media-time terms of Block 5, the two focal interactions (age × Instagram, age × social-media time), loneliness, the four demographic covariates interpreted in the Results (age, female gender, household income, never-married), the primary indicator of each contextual model in Table S8 (self-rated religiosity, the NUTS-1 and urbanization joint F tests, lifetime unemployment experience, sleep duration, maternal emotional closeness), and the two exploratory tests of Table S9; total internet time, education, household size, the three secondary age × platform interactions, the secondary indicators within the religiosity and maternal models (importance of God, prayer frequency; mother-directed video, phone, and text contact), the female × Instagram term, and descriptive or quantile analyses were not included. Extending the family to all 32 candidate coefficient tests in these models leaves every significant test significant (largest corrected p = .046). Tests are drawn from the Block-5 headline model, the contextual models of Table S8, and the exploratory models of Table S9. All twenty raw p-values and the correction are reproducible from the public-use data with the model specifications given in the Methods and Supplementary Material.

**Table S17. Instagram conditional slopes and Johnson–Neyman bounds with and without the loneliness covariate (nine-item psychological-distress index, N = 1,478)**

| **Quantity** | **Block 4 (loneliness not entered)** | **Block 5 (loneliness adjusted)** |
| --- | --- | --- |
| Instagram slope at age 18, B (SE), p | +0.339 (0.118), p = .004 | +0.338 (0.100), p < .001 |
| Instagram slope at age 25 (M - 1 SD), B (SE), p | +0.286 (0.095), p = .003 | +0.277 (0.080), p < .001 |
| Instagram slope at the mean age of 39.53, B (SE), p | +0.166 (0.063), p = .008 | +0.139 (0.055), p = .011 |
| Instagram slope at age 54 (M + 1 SD), B (SE), p | +0.047 (0.083), p = .571 | +0.001 (0.076), p = .992 |
| Age × Instagram interaction, β (p) | -.060 (.059) | -.069 (.014) |
| Johnson–Neyman region of significance (years) | 18.0 to 44.7 | 18.0 to 42.8 |
| Age at which the point estimate reaches zero (years) | 60.3 | 54.5 |
| Model R² | .127 | .359 |

Note. Both models were estimated on the analytical sample with HC3 robust standard errors and all continuous predictors mean-centered, so each block's Instagram coefficient in Table 3 is the conditional slope at the sample mean age of 39.53 years (SD = 14.88). Block 4 comprises the six demographic controls, the two exposure measures, the four platform-frequency indicators and the five age × platform interactions; Block 5 adds the single-item loneliness covariate. Unstandardized slopes are points on the 9-to-36 distress composite per one-unit rise in Instagram frequency. Conditional-slope standard errors were computed from each model's HC3 covariance matrix of the Instagram and age × Instagram coefficients, and the Johnson–Neyman bounds are the ages at which the conditional slope equals its two-tailed .05 critical value on that model's residual degrees of freedom (1,460 in Block 4 and 1,459 in Block 5). Bounds are reported within the observed age range of 18 to 84 years, so a lower bound of 18.0 is the floor of that range rather than an interior crossing. Because the Block-4 interaction term does not clear .05, the Block-4 bounds describe where the conditional Instagram slope is significant rather than moderation established at the conventional threshold. Table 4 and Figures 4 and 5 report the Block-5 model, and loneliness-free age-stratified estimates are reported separately in Table S2. The conditional slopes are re-expressions of the Table 3 coefficients rather than additional tests; the Block-5 age × Instagram term itself enters the twenty-test FDR family in Table S16.
